# Design and synthesis of stable indigo polymer semiconductors for organic field-effect transistors with high fluoride sensitivity and selectivity†

**DOI:** 10.1039/c9ra04302k

**Published:** 2019-08-21

**Authors:** Jenner H. L. Ngai, George Y. Chang, Xiguang Gao, Xiaocheng Zhou, Arthur D. Hendsbee, Yuning Li

**Affiliations:** Department of Chemical Engineering, Waterloo Institute of Nanotechnology, (WIN), University of Waterloo 200 University Ave West Waterloo N2L 3G1 Canada yuning.li@uwaterloo.ca +1-519-888-4347 +1-519-888-4567 ext. 31105

## Abstract

We report the design and synthesis of two novel indigo donor–acceptor (D–A) polymers, PIDG-T-C20 and PIDG-BT-C20, comprising an indigo moiety that has intramolecular hydrogen-bonds as the acceptor building block and thiophene (T) and bithiophene (BT) as the donor building block, respectively. PIDG-T-C20 and PIDG-BT-C20 exhibited characteristic p-type semiconductor performance, achieving hole mobilities of up to 0.016 and 0.028 cm^2^ V^−1^ s^−1^, respectively, which are highest values reported for indigo-based polymers. The better performing PIDG-BT-C20 was used for the fabrication of water-gated organic field-effect transistors (WGOFETs), which showed excellent stability at ambient conditions. The PIDG-BT-C20-based WGOFETs exhibited rapid response when fluoride ions were introduced to the water gate dielectric, achieving a limit of detection (LOD) of 0.40 mM. On the other hand, the devices showed much lower sensitivities towards other halide ions with the order of relative response: F^−^ ≫ Cl^−^ > Br^−^ > I^−^. The high sensitivity and selectivity of PIDG-BT-C20 to fluoride over other halides is considered to be realized through the strong interaction of the hydrogen atoms of the N–H groups in the indigo unit with fluoride ions, which alters the intramolecular hydrogen-bonding arrangement, the electronic structures, and thus the charge transport properties of the polymer.

## Abstract 2

A p-type indigo polymer semiconductor is developed for water-gated organic field-effect transistors (WGOFET) for sensing fluoride ions.

## Graphical abstract

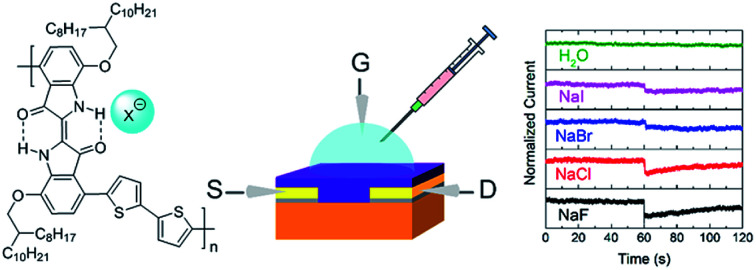

## Introduction

Fluoride (F^−^) is an important component in mammalian biological systems. The uptake of a small amount of fluoride ions in drinking water by humans can strengthen bones and prevent osteoporosis or tooth decay.^1–3^ However, an excess of fluoride ion uptake can lead to dental and skeletal diseases such as fluorosis, osteosarcoma and nephrolithiasis.^4^ Therefore, maintaining the fluoride concentration in drinking water within a proper range is very important for public health.^5,6^ Existing fluoride sensors rely mainly on a potentiometric working principle with ion selective electrodes (ISE) with the aid of a reference electrode for signal (potential) measurement. Other fluoride sensors under development are colorimetric or fluorescence-based optical chemosensors in which the qualitative and quantitative analyses of fluoride ions are achieved with a UV-Vis spectrophotometer or a fluorimeter. The mechanisms of these optical fluoride sensors involve the interactions between the fluoride ion and the sensing chromophore, which affect the intramolecular charge transfer (ICT) or excited state intramolecular proton transfer (ESIPT) of hydrogen-bonds,^7–12^ cleavage of silicon–oxygen or silicon–carbon bonds and binding of fluoride to amides,^13–17^ π–π interactions,^18^ and aggregation of nanoparticles.^19–23^

Printed organic field-effect transistors (OFETs) based on small molecule or polymer semiconductors have drawn much attention in recent years because of their low fabrication cost, excellent substrate conformity, high mechanical robustness and versatile function tunability of the organic semiconductors. Therefore, OFETs have many potential applications such as flexible displays, radio frequency identification tags, chemical or biological sensors and therapeutic medical devices.^24–32^ Nonetheless, although OFETs have been studied as sensors for the detection of numerous chemical analytes,^30,33–37^ there are only a few for sensing ions in aqueous solutions mainly due to the instability of most organic semiconductors towards water and oxygen under device operation. To the best of our knowledge, there has been only one report on the OFET-based fluoride ion sensors,^38^ where the gate electrode instead of the polymer semiconductor layer was in direct contact with the aqueous solution containing the analyte fluoride ions.

**Scheme 1:**
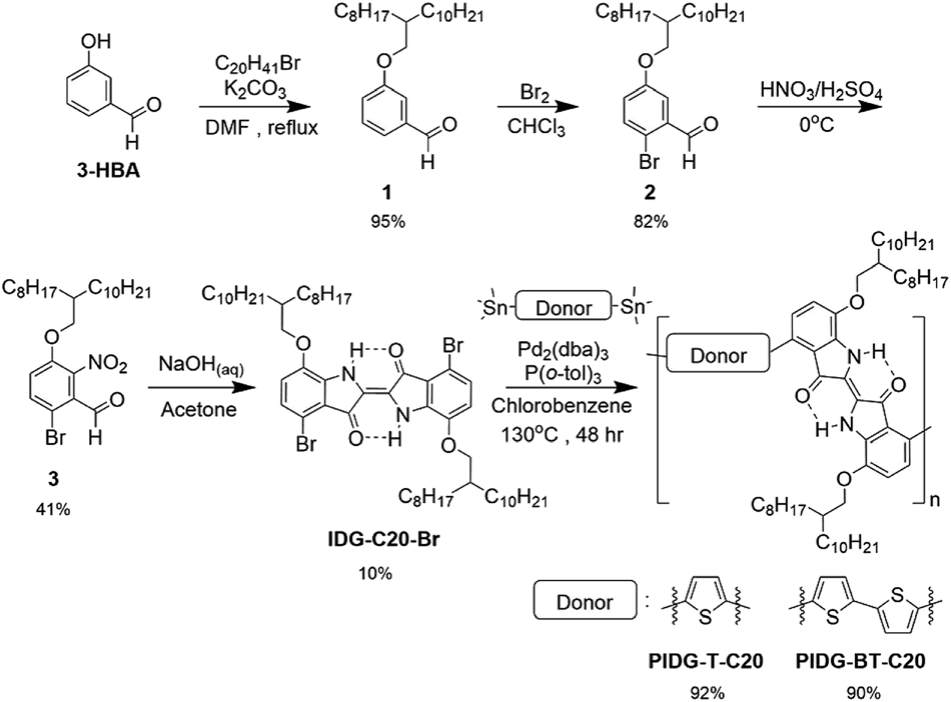
General synthetic scheme of indigo D–A polymers.

Indigo is a stable dyestuff, which has been used for textiles for centuries. Indigo and its small molecule^39–44^ and polymer^45–48^ derivatives have recently demonstrated promising semiconductor properties as channel materials in OFETs. We are particularly interested in the intramolecular hydrogen bonds between the N–H and C=O groups of the two vinylogous amides of indigo, N–H⋯O=C, which may preferentially interact with fluoride ions.^7–12^ In this work, we prepared two novel indigo donor–acceptor (D–A) polymers, PIDG-T-C20 and PIDG-BT-C20 (Scheme 1), comprising the hydrogen-bond-containing indigo as the acceptor building block and thiophene (T) or bithiophene (BT) as the donor building block, respectively. PIDG-T-C20 and PIDG-BT-C20 exhibited p-type semiconductor performance when used as the active layer in bottom-gate-bottom-contact (BGBC) OFETs, achieving hole mobilities of up to 0.016 and 0.028 cm^2^ V^−1^ s^−1^, respectively, which are so far the highest values reported for indigo-based polymers.^46,48,49^ The better performing PIDG-BT-C20 was chosen for fabricating WGOFET devices, which showed excellent stability at ambient conditions. When fluoride ions were introduced to the water gate dielectric, a rapid decrease in the drain current (*I*_DS_) was observed, achieving a limit of detection (LOD) of 0.40 mM for fluoride ions. On the other hand, the devices showed much lower sensitivities towards other halide ions with the order of relative response: F^−^ ≫ Cl^−^ > Br^−^ > I^−^, indicating the excellent selectivity of these sensors to fluoride ions.

## Results and discussion

### Synthesis of indigo-based D–A polymer semiconductors

As afore-mentioned, preserving the intramolecular hydrogen bonds, N–H⋯O=C, for the fluoride sensing is critical in the design of our indigo-based fluoride OFET sensors. However, indigo monomers without solubilizing groups such as Tyrian purple ((*E*)-6,6′-dibromo-[2,2′-biindolinylidene]-3,3′-dione) are insoluble, posing challenges to its purification and polymerization. Therefore, we synthesized a soluble indigo monomer IDG-C20-Br (Scheme 1), which has solubilizing 2-octyldodecyloxy groups at the 7,7′-positions with the hydrogen bounds intact, following a recently developed synthetic route.^39^ Briefly, alkoxylation of 3-hydroxybenzaldehyde (3-HBA) formed 1, which was brominated to afford 2, followed by nitration to give 3. The Baeyer–Drewsen indigo synthesis using 3 was then carried out to give the indigo monomer IDG-C20-Br.^50^ Stille coupling polymerization was carried out between IDG-C20-Br and 2,5-bis(trimethylstannyl)thiophene or 5,5′-bis(trimethylstannyl)-2,2′-bithiophene to produce two polymers PIDG-T-C20 and PIDG-BT-C20, respectively, which were purified by Soxhlet extraction. The molecular weights of the polymers were measured by high temperature gel permeation chromatography (HT-GPC) at 140 °C using 1,2,4-trichlorobenzene (TCB) as eluent. PIDG-T-C20 and PIDG-BT-C20 have number molecular weights (*M*_n_) of 21.6 and 18.6 kDa with dispersities (*Đ*) of 2.57 and 2.08, respectively. Thermogravimetric analysis (TGA) were performed on PIDG-T-C20 and PIDG-BT-C20, which showed good thermal stability with a 5% weight loss temperature at 315 and 361 °C, respectively. No noticeable endo- or exothermic transitions were found on their differential scanning calorimetry (DSC) thermograms (ESI†) up to 250 °C.

### Optical properties and electrochemical properties

**Fig. 1:**
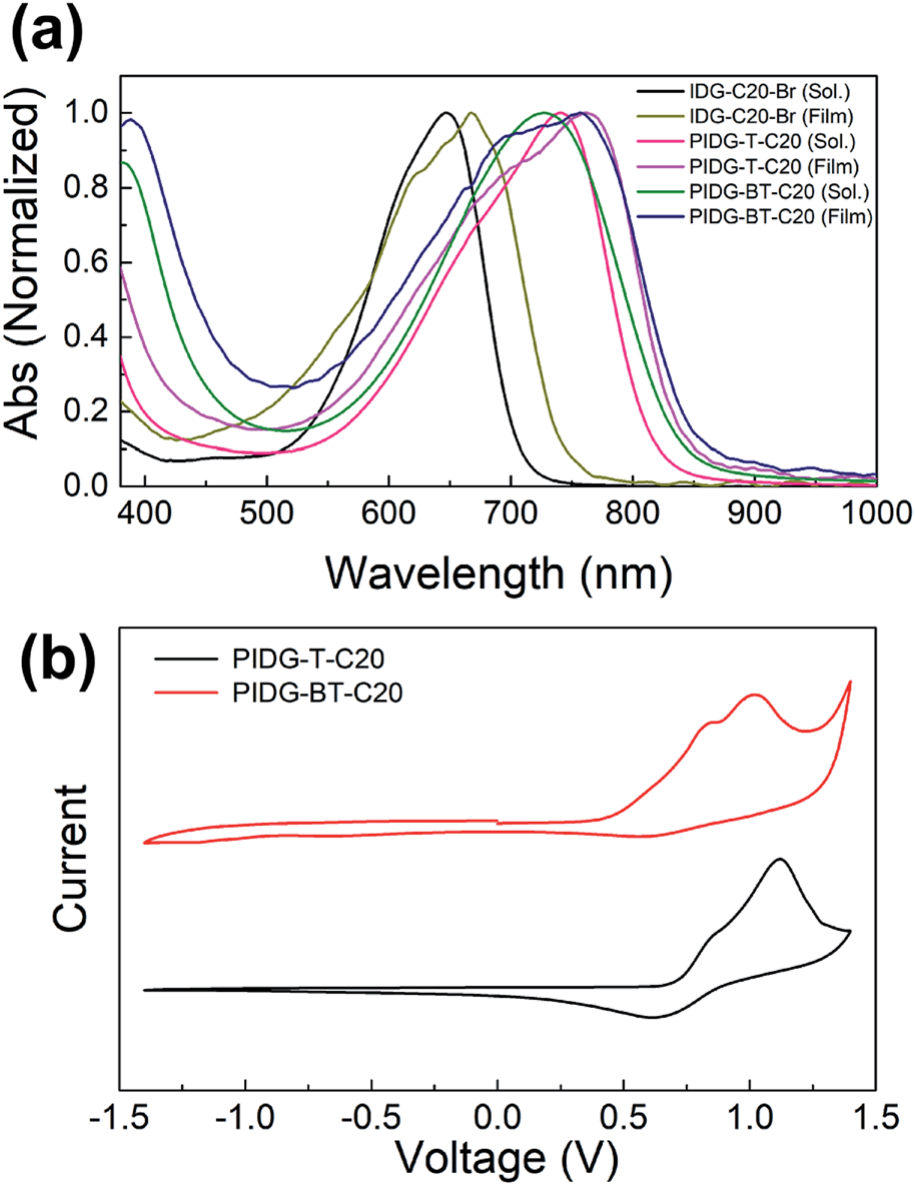
(a) UV-Vis absorption spectra of indigo monomers and polymers chloroform solutions and thin films. (b) Cyclic voltammograms of indigo polymers.

As shown in Fig. 1a and Table 1, PIDG-T-C20 and PIDG-BT-C20 in chloroform solutions exhibited notable red-shifts in the wavelength of maximum absorbance (*λ*_max_ = 741 nm for PIDG-T-C20 and 728 nm for PIDG-BT-C20) when compared to the indigo monomer IDG-C20-Br (*λ*_max_ = 668 nm). This indicated that the polymers have more extended π-conjugation than the indigo monomer. In thin films, they exhibited broader and further red-shifted absorption spectra (*λ*_max_ = 762 nm for PIDG-T-C20 and 758 nm for PIDG-BT-C20), which could be attributed to the planarization of polymer backbone and intermolecular interaction in the solid state. Cyclic voltammetry (CV) diagrams of PIDG-T-C20 and PIDG-BT-C20 showed oxidative peaks, which were used to calculate their HOMO energy levels to be −5.48 and −5.27 eV, respectively (Fig. 1b and Table 1). On the other hand, no noticeable reduction peaks were observed for both polymers. Therefore, their LUMO energy levels were calculated using the obtained HOMO energy levels and the optical band gaps to be −4.00 eV and −3.80 eV for PIDG-T-C20 and PIDG-BT-C20, respectively.

**Table 1:** UV-Vis optical and electrochemical information of indigo polymer films

Polymer	*E*_g,opt_ (eV)	*λ*_max_ (nm)	*λ*_onset_ (nm)	HOMO_CV_ (eV)	LUMO_opt/CV_ (eV)
PIDG-T-C20	1.48	762	839	−5.48	−4.00
PIDG-BT-C20	1.47	758	844	−5.27	−3.80

### OFET performance of polymers

**Fig. 2:**
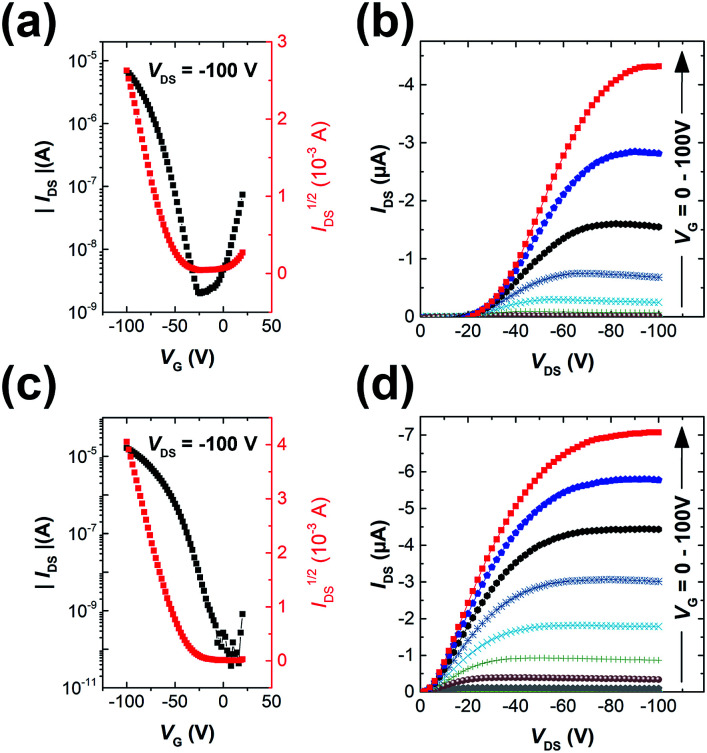
Transfer characteristics of (a) PIDG-T-C20 and (c) PIDG-BT-C20 films annealed at 150 °C; output characteristics of (b) PIDG-T-C20 and (d) PIDG-BT-C20 films annealed at 150 °C with *V*_G_ step voltage = −10 V.

The polymers were used as channel semiconductors in BGBC OFETs. Both polymers exhibited typical p-type semiconductor characteristics with the maximum hole mobilities of up to 0.016 cm^2^ V^−1^ s^−1^ for PIDG-T-C20 and 0.028 cm^2^ V^−1^ s^−1^ for PIDG-BT-C20 for films annealed at 150 °C (Fig. 2 and Table 2). It is noticed that the devices based on PIDG-T-C20 showed obvious S-shaped output curves near the origin, indicating the existence of large contact resistances. This might be due to its rather low HOMO energy level that builds up a large hole injection barrier as well as its poor contact with the source/drain electrodes. All the devices exhibited rather large threshold voltages (*V*_th_ = *ca.* −30 to −60 V), indicating the presence of a large number of hole traps. A representative PIDG-BT-C20-based OFET device showed a hysteresis Δ*V*_th_ (the difference between forward and reverse) of 7.9 V (Fig. S7 in ESI†), which is within the typical range for OEFTs.^51–54^ However, the PIDG-T-C20-based OFET device exhibited a quite large Δ*V*_th_ of 30.9 V, which might be caused by the trap recharging that originated from the large grain boundary resistance.^53,55^

**Table 2:** OFET device data for polymer PIDG-T-C20 and PIDG-BT-C20

Polymer	Annealing temp. (°C)	Avg. ± Std *μ*_sat_ (cm^2^ V^−1^ s^−1^)	Max. mobility (cm^2^ V^−1^ s^−1^)	*V*_th_ (V)	*I*_ON/OFF_
PIDG-T-C20	50	0.0024 ± 0.00093	0.0036	−34.2	1.3 × 10^4^
	100	0.0050 ± 0.00098	0.0059	−58.8	3.1 × 10^4^
	150	0.015 ± 0.00052	0.016	−52.0	3.0 × 10^3^
	200	0.0022 ± 0.00067	0.0029	−63.7	3.0 × 10^3^
PIDG-BT-C20	50	0.0020 ± 0.00034	0.0025	−43.3	1.7 × 10^4^
	100	0.00027 ± 0.00011	0.00037	−39.9	1.3 × 10^3^
	150	0.027 ± 0.00091	0.028	−43.8	1.4 × 10^5^
	200	0.0030 ± 0.00039	0.0035	−43.6	3.3 × 10^4^

### Morphology and crystallinity of polymer films

**Fig. 3:**
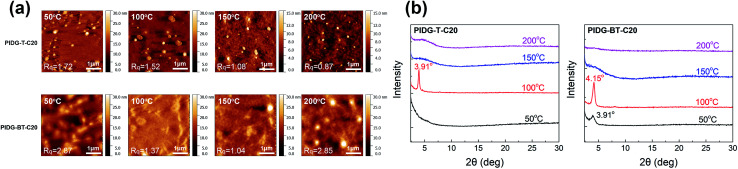
(a) AFM images of PIDG-T-C20 and PIDG-BT-C20 annealed at 50, 100, 150, and 200 °C. (b) XRD spectra of PIDG-T-C20 and PIDG-BT-C20.

The atomic force microscopic (AFM) image of the PIDG-T-C20 film annealed at 50 °C showed some micron-sized particles (bright spots) on the rather smooth film surface (*R*_q_ = 1.7 nm) (Fig. 3a). Upon annealing at 100 °C, fewer but larger particles were seen. At 150 °C, both the number and size of the particles decreased along with a decrease in the film roughness (*R*_q_ = 1.1 nm), which may be accounted for the optimum hole mobility achieved at this annealing temperature. Further increasing the annealing temperature to 200 °C led to the formation of more clearly defined aggregates along with larger and deeper cracks. This might create discontinuous regions, resulting in a decrease in mobility. Compared to PIDG-T-C20, the 50 °C-annealed PIDG-BT-C20 film showed larger spherical and rod-like aggregates with a higher roughness (*R*_q_ = 2.9 nm). The film became smoother by increasing the annealing temperature to 100 °C (*R*_q_ = 1.4 nm) and 150 °C (*R*_q_ = 1.1 nm), which might be accounted in part for the improved carrier mobility of PIDG-BT-C20. When the film was annealed at 200 °C, larger aggregates with clearer grain boundaries formed, which could explain the significant drop in mobility of this polymer at this annealing temperature.

The crystallinity of the polymer thin films was studied by using XRD in the reflection mode. For the PIDG-T-C20 film annealed at 50 °C, no diffraction peak was observed, indicating a quite disordered chain packing of this polymer. Upon annealing at 100 °C, a peak at 2*θ* = 3.91° appeared, which corresponds to a *d*-spacing of 22.6 Å (Fig. 3b). This peak could be assigned to the (100) diffraction, representing the interlamellar distance. The sole appearance of this peak also suggested that the polymer chains adopted an edge-on orientation, which is favourable for charge transport in OFETs.^56–58^ For PIDG-BT-C20, a (100) peak at 2*θ* = 3.91° (*d* = 22.6 Å) was observed at a lower annealing temperature of 50 °C (Fig. 3b). The peak intensified and shifted to 4.15° (*d* = 21.3 Å) upon annealing at 100 °C, which indicates a closer interlamellar packing distance. For both polymers, the (100) peak disappeared at annealing temperatures higher than 150 °C, suggesting that the polymer chains might have undergone re-organization to form more disordered chain packing. Although both polymers exhibited maximum crystallinity at the annealing temperature of 100 °C, their highest hole mobilities in OFETs were obtained at the annealing temperature of 150 °C where both polymers were disordered. It has been reported that the film morphology may play a more significant role in charge transport than the crystallinity of the polymer films.^59^ Therefore, the observed optimal charge transport performance for the 150 °C-annealed films of both polymers may be due to their lower surface roughness as observed in the AFM images at this annealing temperature.

### Halide ion sensing properties of PIDG-BT-C20-based WGOFET

**Fig. 4:**
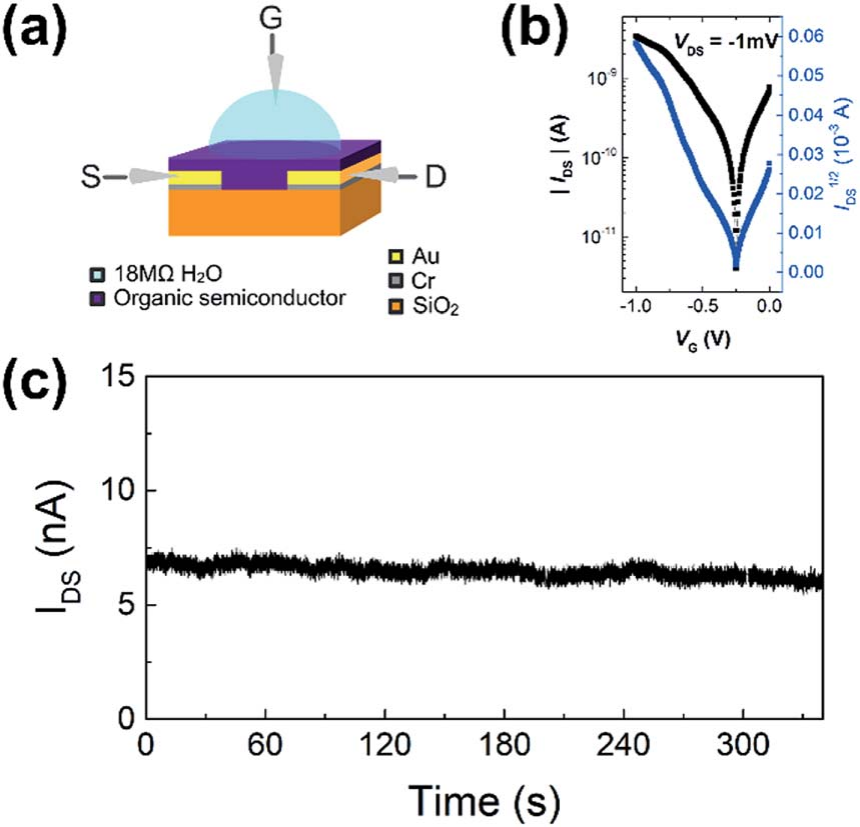
(a) Schematic device structure of a WGOFET with a 20 μL 18 MΩ DI water droplet sitting on top of the active later as gate dielectric. (b) WGOFET p-type transfer characteristic of PIDG-BT-C20. (c) Current *versus* time graph of PIDG-BT-C20 WGOFET operated at *V*_DS_ = −1 mV and *V*_G_ = −1 V for 340 seconds.

WGOFETs were fabricated on the bare Si/SiO_2_ substrate having interdigitated source and drain electrodes with channel length (*L*) of 30 μm and channel width (*W*) of 15.8 mm (Fig. 4a). PIDG-BT-C20 was chosen as the active layer for WGOFETs because it showed better OFET performance than PIDG-T-C20. For the transistor measurement, 20 μL of 18 MΩ deionized (DI) water was dropped on top of the active layer *via* a micropipette and a probe needle as the gate electrode was connected to the top of the water droplet. Since electrolysis of water would start to occur at a potential difference of 1.23 V,^60^ both the gate (*V*_G_) and source-drain (*V*_DS_) voltages were kept below an absolute value of 1.23 V. The transfer characteristics of the PIDG-BT-C20 WGOFET devices at *V*_DS_ = −1 mV and *V*_G_ = 0 to −1.0 V measured in air are shown in Fig. 4b. A stable signal baseline (measured without analyte) is an important criterion for sensors. As shown in Fig. 4c, the *I*_DS_ of the PIDG-BT-C20 WGOFET device remained relatively steady over time, indicating the excellent stability of this polymer towards water and air. The baseline was found to remain steady after repeated measurements and washing with water multiple times. A reference WGOFET device using regioregular head-to-tail poly(3-hexylthiophene) (P3HT) as the active layer was also fabricated and characterized as a comparison. However, the P3HT based WGOFET device showed significant deviations in *I*_DS_ over time (Fig. S8†) and started to degrade a few hours after the measurement. The much better stability of PIDG-BT-C20 is considered due to its lower HOMO energy level (−5.27 eV) than that of P3HT (*ca.* −5.0 eV).^61,62^

**Fig. 5:**
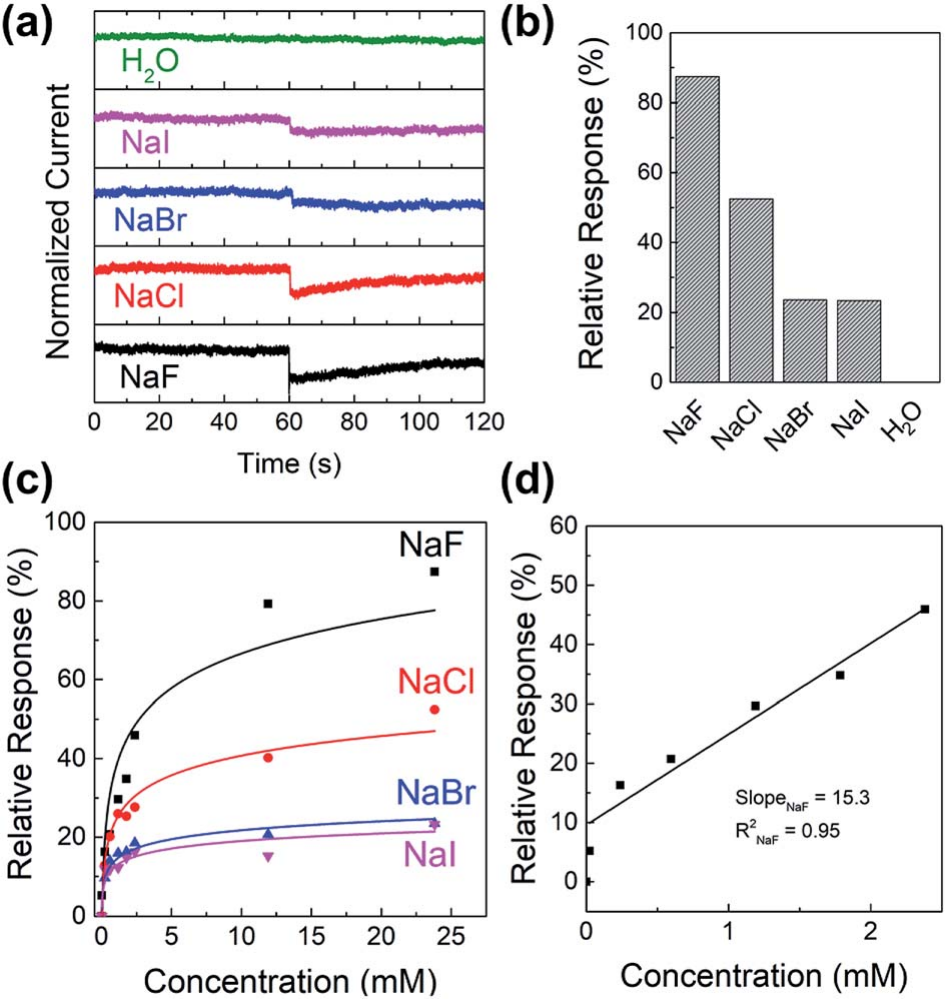
(a) Current *versus* time graph of sodium halide sensing experiment where 5 μL of 24 mM analyte (NaF, NaCl, NaBr, NaI or pure water) was injected into the water-gate droplet at around time = 60 s; (b) histogram showing the relative response of 24 mM halide ion sensing of the PIDG-BT-C20 WGOFET sensor obtained in (a); (c) relative response *versus* concentration of analyte graph of sodium halides; (d) linear region of relative response *versus* concentration of NaF and a best-fitted calibration curve of NaF.

To study the sensitivity and selectivity of the PIDG-BT-C20 WGOFET devices towards F^−^ over other halide ions (Cl^−^, Br^−^, and I^−^), a series of aqueous solutions of various sodium halides (NaF, NaCl, NaBr, and NaI) with different concentrations were used as analytes. As shown in Fig. 5a, the device with 20 μL DI water as the dielectric was first operated at *V*_DS_ = −1 mV and *V*_G_ = −1 V. After 60 s, 5 μL of DI water or sodium halide aqueous solution was injected into the water-gate dielectric. Injection of DI water did not cause any change in the drain current, while all halide solutions caused an immediate current drop. The device exhibited the largest current drop when the NaF solution was injected, indicating the highest sensitivity of the device towards the F^−^ ions. The differences in drain current before and after halide ion introduction were used to calculate the relative response (*S*) of the device according to the following eqn (1):1
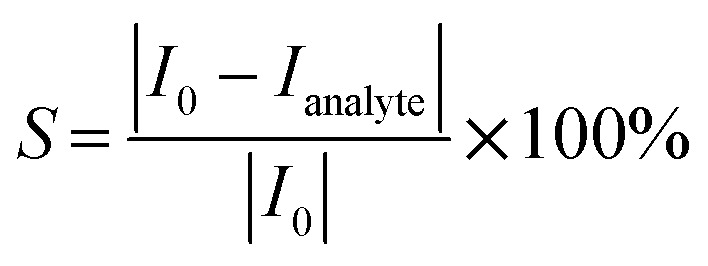
where *I*_0_ is the *I*_DS_ of the baseline and *I*_analye_ is the *I*_DS_ after analyte introduction. The relative responses (*S*) for 24 mM NaF, NaCl, NaBr, and NaI solutions were 87%, 52%, 23%, and 22% (Fig. 5b), demonstrating the excellent selectivity of this device towards the F^−^ ions over other halides.

Fig. 5c shows the relative response (*S*) of sensing sodium halides with varying halide concentrations, which could be a demonstration of using the sensor device for quantitative analysis of halide solutions. A response saturation in the higher concentration range was observed. This is possibly because of the sensor-analyte association-dissociation kinetics had reached an equilibrium state,^63^ and is a typical observation for chemical sensors.^64–67^Fig. 5d shows the linear regression of S *vs.* [NaF] in the low NaF concentration range of 0–2.4 mM. The slope and coefficient of determination (*R*^2^) of the calibration curve was found to be 15.3 and 0.95, respectively. The limit of detection (LOD) was calculated using the eqn (2):^68^2
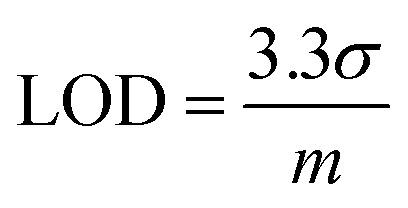
where, *σ* is the relative standard deviation of the sensitivity plot of the device in the absence of analyte and *m* is the slope of the analyte calibration curve. The LOD for fluoride (NaF) was found to be 0.40 mM for the PIDG-BT-C20 WGOFET device, which is better than the LOD (0.7 mM) of the previously reported OFET-based fluoride ion sensors.^38^

### Study of halide ion sensing mechanism of polymers

**Fig. 6:**
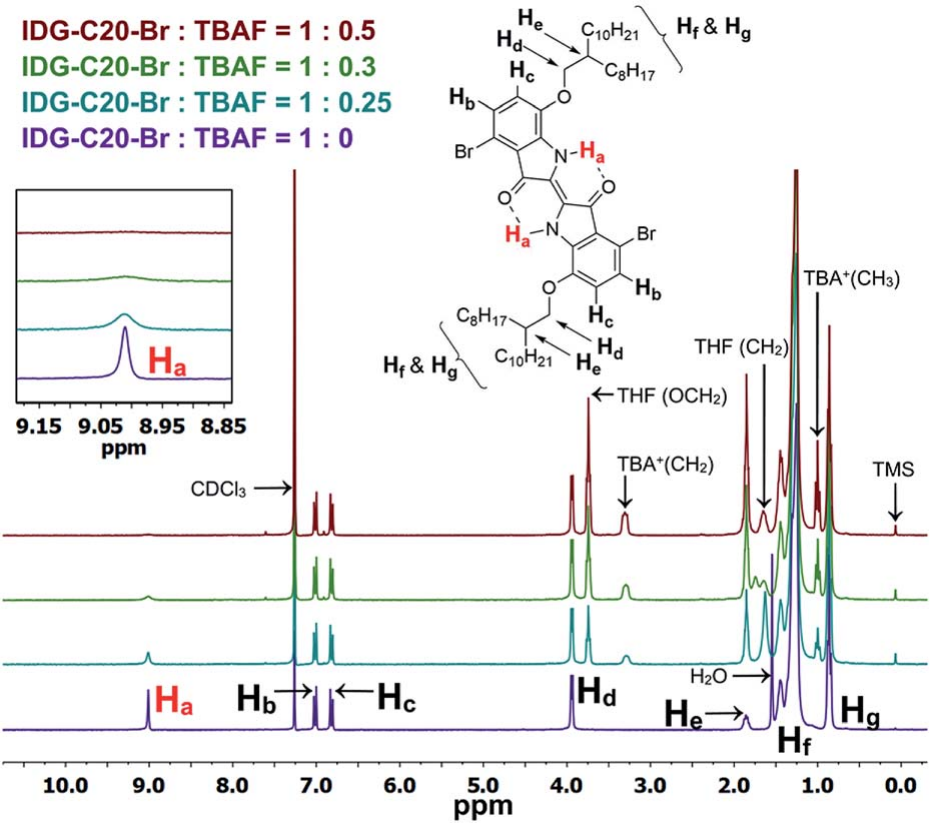
^1^H NMR titration of IDG-C20-Br with TBAF (0–0.5 equiv.) in CDCl_3_.

As previously mentioned, the design principle for these indigo-based polymers is to utilize the intramolecular hydrogen bonding of indigo amide N–H⋯O=C to recognize the fluoride ions. Since fluorine has the highest electronegativity among all elements, the N–H⋯F^−^ interaction is expected to be the strongest among all halides. The interaction of the amide hydrogen bonds on PIDG-BT-C20 with fluoride ions may be similar to that of some previously reported host-guest supramolecular fluoride chemosensors using amide receptors.^69–72^^1^H NMR titration^71–73^ was adopted as an effective method to verify the N–H⋯F^−^ interactions. The small molecule IDG-C20-Br was used as the model compound since the ^1^H NMR signals of polymer PIDG-BT-C20 were very broad and weak. ^1^H NMR titration was performed using tetrabutylammonium fluoride (TBAF) in CDCl_3_. It was observed that the amide proton peak H_a_ at 9.01 ppm broadened when the ratio of TBAF/IDG-C20-Br was increased from 0 to 0.3 molar equiv. and disappeared at a 0.5 equiv. (Fig. 6), indicating the strong interaction of H_a_ with F^−^.^74,75^ On the other hand, addition of same amounts of tetrabutylammonium chloride (TBAC), bromide (TBAB), or iodide (TBAI) did not result in any observable changes in the H_a_ signal (Fig. S18–S20 in ESI†).

**Fig. 7:**
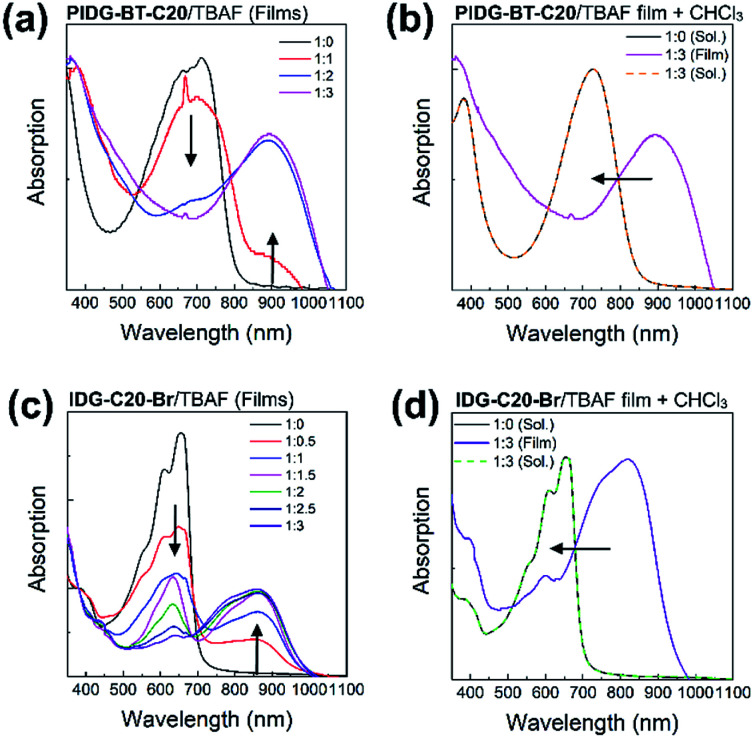
(a) and (c) Absorption spectral changes of thin films samples of PIDG-BT-C20 and IDG-C20-Br upon addition of different TBAF molar ratios (0–3 equivalent). (b) and (d) Normalized pristine PIDG-BT-C20 and IDG-C20-Br solutions in chloroform (black solid line) and the reversible absorption spectral change (dotted lines) by dissolving the thin film sample of PIDG-BT-C20 or IDG-C20-Br : TBAF = 1 : 3 back into chloroform solutions.

UV-Vis spectroscopy was used to further demonstrate the interaction of fluoride ions with the indigo chromophore by measuring the films of PIDG-BT-C20 or IDG-C20-Br blended with 0–3 molar equivalents of TBAF. The peaks representing the π–π* transition at *λ*_max_ = 713 nm for PIDG-BT-C20 and 656 nm for IDG-C20-Br weakened gradually and a new long wavelength absorption band at *λ*_max_ = 894 and 862 nm started to appear and intensified with the increasing amount of TBAF from 0 to 3 equivalents (Fig. 7a and c). No changes in the absorption spectra were observed when TBAC, TBAB, and TBAI were blended into the PIDG-BT-C20 and IDG-C20-Br films, suggesting the much weaker interactions of Cl^−^, Br^−^, and I^−^ with PIDG-BT-C20 and IDG-C20-Br. The significant current decreases observed for WGOFETs as fluoride ions were introduced could be a result of the disruption of hydrogen-bonds in the indigo moiety by fluoride ions (*vide supra*). Interestingly, it was found that when the PIDG-BT-C20/TBAF and IDG-C20-Br/TBAF blended film samples were dissolved back into chloroform, the spectra of the obtained solutions became identical to those of pristine PIDG-BT-C20 or IDG-C20-Br (Fig. 7b and d). This observation indicates that the fluoride-indigo interaction was interrupted in solution due to the solvation of fluoride ions and indigo moieties by the large amounts of solvent molecules.

**Fig. 8:**
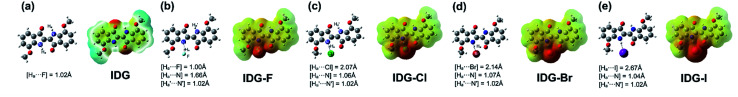
DFT results of (a) indigo model compound IDG and indigo-halide model compounds (b) IDG-F; (c) IDG-Cl; (d) IDG-Br and (e) IDG-I at the B3LYP/3-21G* level of theory.

Furthermore, computer simulations of the interaction between the indigo moiety and halide anions were conducted using density functional theory (DFT) with the B3LYP/3-21G* level under tight convergence. The overall charge was set to be −1 and a singlet spin state was used as the initialization parameters for DFT calculations for the model complexes, IDG-F, IDG-Cl, IDG-Br and IDG-I. The neutral indigo model compound IDG was also simulated as a reference (Fig. 8).

The simulation results show that IDG has an interatomic distance of 1.02 Å for the H_a_⋯N bond. When a fluoride anion was added, the formed complex IDG-F has an increased H_a_⋯N distance of 1.66 Å, while the distance between H_a_ and F^−^, [H_a_⋯F], is very short at 1.00 Å, indicating the formation of a new hydrogen-bond (Fig. 8b). On the other hand, the amide N–H interatomic distance on the opposite side of indigo was not affected by the fluoride anion 
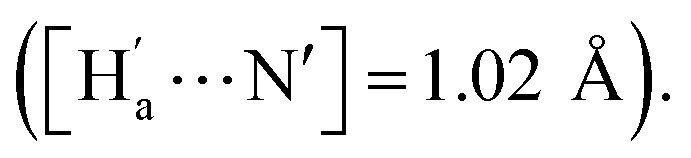
 For other IDG-halide complexes simulated, the H_a_⋯N distances were only slightly affected ([H_a_⋯N] = 1.06 Å for IDG-Cl, 1.07 Å for IDG-Br, and 1.04 Å for IDG-I). A summary of simulation results can be found in Table S1 in the ESI.†

To further demonstrate the importance of the intramolecular amide hydrogen bonding in PIDG-BT-C20 for the observed high sensing selectivity towards fluoride ions, a DPP-based D–A polymer semiconductor, PDQT,^51,76^ which contains amide moieties (with alkyl substituents at nitrogen atoms), but no N–H groups and intramolecular hydrogen bonds, was used as a channel in a WGOFET for halide ion sensing. It was found that the device showed the relative responses in the order of F^−^ < Cl^−^ < Br^−^ < I^−^ (ESI†), which is opposite to that of the PIDG-BT-C20 devices. These results strongly indicate the critical roles of the N–H groups and intramolecular hydrogen bonding in PIDG-BT-C20 played in the observed high sensitivity and selectivity towards fluoride ions for this polymer.

## Conclusion

In this work, two indigo-based donor–acceptor polymers PIDG-T-C20 and PIDG-BT-C20, where the indigo building block has intramolecular hydrogen bonds, were designed and synthesized for use as channel semiconductors in OFETs for fluoride ion sensing. The charge transport performance of PIDG-T-C20 and PIDG-BT-C20 was evaluated in BGBC OFETs, demonstrating highest hole mobilities of up to 0.016 and 0.028 cm^2^ V^−1^ s^−1^, respectively, which are highest values reported for indigo-based polymers. A water-gated organic field-effect transistor was fabricated using PIDG-BT-C20, which exhibited excellent stability at ambient conditions. When a small aliquot of aqueous solution containing F^−^, Cl^−^, Br^−^, or I^−^ was introduced, the device demonstrated halide ions with the order of relative response: F^−^ ≫ Cl^−^ > Br^−^ > I^−^, indicating the excellent selectivity of this sensor to fluoride ions. The limit of detection (LOD) for NaF was calculated to be 0.40 mM, which is better than the previously reported OFET based fluoride sensors. The mechanism of fluoride selectivity of the WGOFET sensor was studied through ^1^H NMR, UV-Vis and computer simulations, which indicated the much stronger interaction of fluoride with the intramolecular hydrogen bond N–H⋯O=C in the indigo unit of the polymer compared with other halides. The disruptive effect of fluoride on the hydrogen bond would alter the electronic structure and thus the charge transport properties of the polymer, leading to the high sensitivity of the device towards fluoride. Our results demonstrated that the intramolecular hydrogen bond-containing indigo polymers are a promising class of the semiconductors for OFET based fluoride sensors, which showed good field-effect transistor performance, excellent stability at ambient conditions, and high sensitivity and selectivity towards fluoride ions. They have the potential to be printed on flexible plastic substrate as a low-cost, portable alternative or replacement to other types of fluoride sensors.

## Conflicts of interest

There are no conflicts to declare.

## Supplementary Material

RA-009-C9RA04302K-s001
