# Supplementary material for: Design and synthesis of stable indigo polymer semiconductors for organic field-effect transistors with high fluoride sensitivity and selectivity
Source: RSC Adv. 2019 Aug 21;9(45):26230–7. doi: 10.1039/c9ra04302k (PMC9070390; doi:10.1039/c9ra04302k)
Supplement: RA-009-C9RA04302K-s001 [file RA-009-C9RA04302K-s001.pdf]

Electronic Supplementary Material (ESI) for RSC Advances.
This journal is © The Royal Society of Chemistry 2019

Supplementary Information

Design and synthesis of stable indigo polymer semiconductors for organic field-effect transistors with high fluoride sensitivity and selectivity

Jenner H.L. Ngai, George Y. Chang, Xiguang Gao, Xiaocheng Zhou, Arthur D. Hendsbee and Yuning Li*

Department of Chemical Engineering and Waterloo Institute of Nanotechnology, (WIN), University of Waterloo, 200 University Ave West, Waterloo, N2L 3G1, Canada. E-mail: yuning.li@uwaterloo.ca; Fax: +1-519-888-4347; Tel: +1-519-888- 4567 ext. 31105

Contents

1. Materials 3
2. Characterizations 3
   2.1. Instruments 3
   2.2. Fabrication of OFET and WGOFET devices 3
3. Synthesis 5
   3.1. Synthesis of indigo monomer 5
      3.1.1. Synthesis of 3-((2-octyldodecyl)oxy)benzaldehyde Compound **1** 5
      3.1.2. Synthesis of 2-bromo-5-((2-octyldodecyl)oxy)benzaldehyde (Compound **2**) 6
      3.1.3. Synthesis of 6-bromo-2-nitro-3-((2-octyldodecyl)oxy)benzaldehyde (Compound **3**) 7
      3.1.4. Synthesis of (*E*)-4,4'-dibromo-7,7'-bis((2-octyldodecyl)oxy)-[2,2'-biindolinylidene]-3,3'-dione (**IDG-C20-Br**) 8
   3.2. Synthesis of polymer **PIDG-T-C20** and **PIDG-BT-C20** 9
4. Additional Data 10
5. Reference 21

1. Materials

All chemicals were purchased from Sigma Aldrich and other commercial sources and used as received. PDQT was synthesized according to previously procedures.[1]

2. Characterizations

2.1. Instruments

UV-Vis spectroscopy measurements were performed on a Thermo Scientific GENESYS™ 10S VIS spectrophotometer. Thermogravimetric analysis (TGA) was carried out on a TA Instruments SDT 2960 at a scan rate of 10 °C min^{-1} under nitrogen. Differential scanning calorimetry (DSC) was carried out on a TA Instruments Q2000 at a temperature ramping rate of 20 °C min^{-1} under nitrogen. High-temperature gel permeation chromatography (HT-GPC) measurements were performed on a Malvern 350 HT-GPC system. Cyclic voltammetry (CV) data was collected on a CHI600E electrochemical analyser using an Ag/AgCl reference electrode and two Pt disk electrodes as the working and reference electrodes in a 0.1 M tetrabutylammonium hexafluorophosphate solution in acetonitrile at a scan rate of 50 mV s^{-1}. Ferrocene was used as the reference, which has a HOMO energy value of -4.8 eV.[2] Nuclear magnetic resonance (NMR) spectrum was obtained using a Bruker DPX 300 MHz spectrometer with chemical shifts relative to tetramethylsilane (TMS, 0 ppm). Matrix-assisted laser desorption/ionization (MALDI) experiment was performed using a Bruker Autoflex Speed MALDI-TOF mass spectrometer using red phosphorus clusters as mass calibration. Reflective X-ray diffraction (XRD) measurements were carried out on a Bruker D8 Advance diffractometer with Cu Ka radiation (l = 0.15406 nm) using polymer films spin coated on SiO_2/Si substrates. Atomic force microscopy (AFM) images were taken with a Dimension 3100 scanning probe microscope. Transistor measurements were performed using an Agilent B2912A Semiconductor Analyser. All computer simulations were carried out by using the Gaussian 09 software.

2.2. Fabrication of OFET and WGOFET devices

The heavily n-doped Si substrate with a 300 nm SiO_2 layer was cleaned by submerging in isopropanol and sonicated for 15 min. The cleaning procedure was repeated with acetone and chloroform. The substrates were then dried with compressed nitrogen and was exposed to O_2 plasma for 2 min. The substrate surface was then modified with a self-assembled monolayer (SAM) by submerging the substrates into a toluene solution of trichlorododecylsilane (DDTS) (~10 mM)

for 20 minutes. The substrates were then washed with toluene and dried with compressed nitrogen. A polymer solution of **PIDG-T-C20** (5mg mL-1 in chloroform) or **PIDG-BT-C20** (5mg mL^{-1} in dichlorobenzene) was spin-coated (3000 rpm) on top of the substrate for 80 s to deposit a 30 nm thick polymer layer. The transistor was then placed on a hotplate and annealed for 20 min at different temperatures (50, 100, 150 or 200 °C) in an argon-filled glovebox prior to the transistor measurements. Carrier mobility was calculated in the saturation regime according to the following equation:

$$I_{DS} = \left(\frac{WC_i}{2L}\right)\mu(V_G - V_T)^2$$

where I_{DS} is the source-drain current, μ is the charge carrier mobility, C_i is the capacitance per unit area of the dielectric (11.6 nF cm^{-2} for DDTS modified SiO_2), W (1000 μm) and L (30 μm) are OTFT channel width and length, V_G is the gate voltage, and V_T is the threshold voltage.

The fabrication of WGOFETs were similar to that of OFETs except bare SiO_2 was used instead of DDTS modified SiO_2 transistor substrates and a larger channel width (W) of 15.8 mm was used. A water droplet was then added on top of the semiconductor layer as the gate dielectric and a probe tip was positioned on top of the water droplet as the gate electrode. The WGOFET performance and stability of P3HT WGOFET device are shown in Figure S8. The general procedure for analyte sensing using WGOFET is depicted in Figure S9. The PDQT sensing experiment for different sodium halides can be seen in Figure S10.

3. Synthesis

3.1. Synthesis of indigo monomer

3.1.1. Synthesis of 3-((2-octyldodecyl)oxy)benzaldehyde Compound **1**

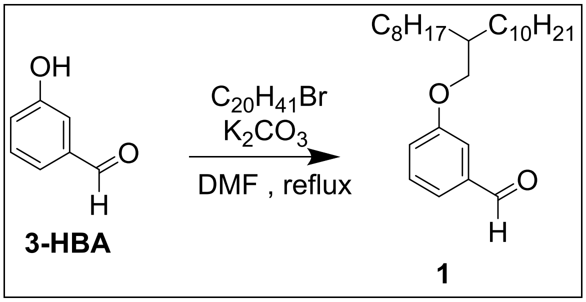

To a 500 mL round-bottom flask, 3-hydroxybenzaldehyde (3-HBA) (10 g, 0.082 mol) was dissolved in dimethylformamide (150 mL). Potassium carbonate (22.6 g , 0.164 mol) was added to the solution of 3-HBA. 9-(Bromomethyl)nonadecane (17.4 g , 0.090 mol) was added in one portion to the reaction mixture. The mixture was equipped with a condenser and was heated to reflux for 18 h under nitrogen. The reaction mixture was cooled down to room temperature, and water and hexane were added to the mixture. The solution was then stirred at room temperature until the excess potassium carbonate powder was dissolved in the aqueous layer. The crude product was extracted with hexane and washed with water and saturated NaCl solution. The organic layer was collected and anhydrous Na_2SO_4 was added to remove residual water in the organic solution. The solution was filtered and solvent was removed by rotary evaporation. The crude product was then purified by a short silica column using pure hexane as eluent to afford compound **1** as a colorless liquid (18.2 g, 95%). ^{1}H NMR (300 MHz, Chloroform-*d*) δ 9.97 (s, 1H), 7.43 (d, J = 4.9 Hz, 2H), 7.39 (s, 1H), 7.17 (m, 1H), 3.89 (d, J = 5.6 Hz, 2H), 1.98 (t, J = 7.8 Hz, 1H), 1.57 – 1.10 (m, 27H), 0.88 (t, J = 6.5 Hz, 6H). ^{13}C NMR (300 MHz, Chloroform-*d*) δ 192.25, 159.98, 137.77, 129.93, 123.21, 121.99, 112.84, 71.23, 37.92, 31.92, 31.34, 30.00, 29.65, 29.35, 26.84, 22.69, 14.11. (Figure S11 & S12)

3.1.2. Synthesis of 2-bromo-5-((2-octyldodecyl)oxy)benzaldehyde (Compound **2**)

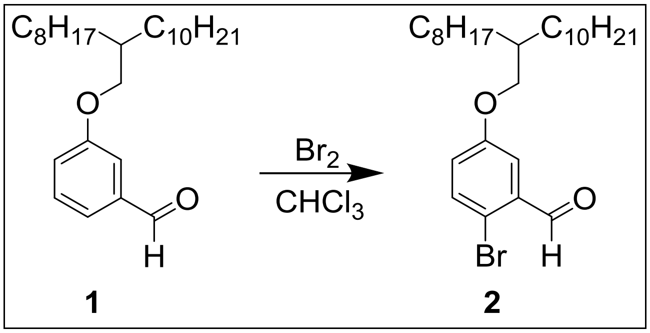

To a two-necked 500 mL round-bottom flask, compound **1** (6.0 g, 0.015 mol) was dissolved in chloroform (100 mL). A solution of bromine (0.92 mL, 0.018 mol) in chloroform (10 mL) was added dropwise into the reaction mixture with a syringe through the rubber septum in 20 min. The reaction mixture was then stirred for an additional 40 min. Cold water was added to the mixture followed by sodium bisulfite solution to eliminate the unreacted bromine. The reaction mixture was then extracted with hexane and washed with water and saturated NaCl solution. The organic layer was collected and anhydrous Na_2SO_4 was added to remove residue of water in the organic solution. The solution was filtered, and solvent was removed by rotary evaporation. The crude product was then purified by a short silica column using pure hexane as eluent to afford compound **2** as a colorless liquid (5.9 g, 82%). ^{1}H NMR (300 MHz, Chloroform-*d*) δ 10.31 (s, 1H), 7.51 (d, *J* = 8.8 Hz, 1H), 7.40 (d, *J* = 3.1 Hz, 1H), 7.03 (dd, *J* = 8.8, 3.2 Hz, 1H), 3.85 (d, *J* = 5.6 Hz, 2H), 1.94 – 1.68 (m, 1H), 1.46 – 1.09 (m, 32H), 0.88 (t, *J* = 6.6 Hz, 6H). ^{13}C NMR (300 MHz, Chloroform-*d*) δ 191.92, 159.07, 134.45, 123.50, 113.42, 71.52, 37.85, 31.92, 31.28, 29.98, 29.66, 29.57, 29.35, 26.81, 22.69, 14.12. (Figure S13 & S14)

3.1.3. Synthesis of 6-bromo-2-nitro-3-((2-octyldodecyl)oxy)benzaldehyde (Compound **3**)

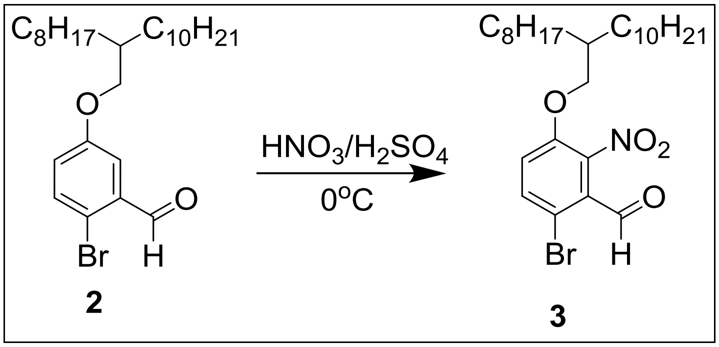

To a 100 mL round-bottom flask, 65% nitric acid (1.40 mL, 0.0201 mol) was slowly added to 96% concentrated sulfuric acid (18 mL, 0.334 mol) at 0 °C and the mixture was stirred for 10 min to form the nitrating reagent. Compound **2** (7.5 g , 0.0155 mol) was added dropwise to the nitrating reagent in 10 min. The solution was kept stirring at 0 °C for 30 min. Crushed ice was added directly to the reaction mixture to precipitate the nitration product. The crude product was collected by suction filtration and washed with cold water until the filtrate is neutral as monitored by pH paper. The crude product was recrystallized in acetone/water solution and dried in vacuum at room temperature to obtain a yellow sticky paste of compound **3** (3.3 g, 41%). The crude product was used directly in the next step without further purification.

3.1.4. Synthesis of (*E*)-4,4'-dibromo-7,7'-bis((2-octyldodecyl)oxy)-[2,2'-biindolinylidene]-3,3'-dione (**IDG-C20-Br**)

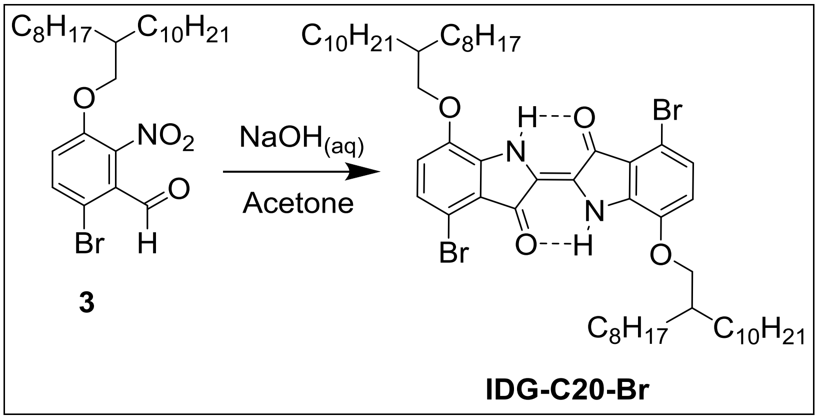

To a 100 mL two-necked round-bottom flask, compound **3** (7 g, 0.0133 mol) was dissolved in acetone (105 mL). One neck of the flask was connected to argon atmosphere and the other neck was stoppered with a rubber septum. The mixture was stirred in an ice bath in argon atmosphere for 10 min. A solution of 0.2 N sodium hydroxide (14 mL) solution was added slowly to the acetone solution with a syringe through the rubber septum at moderate stirring. When color of the solution turned deep red, a 0.4 N sodium hydroxide solution (105 mL) was added slowly. The reaction mixture was stirred for another 15 min. The temperature of the reaction mixture was then raised to room temperature. When the mixture turned deep green, the temperature of the reaction mixture was further increased to refluxing temperature and stirred for an additional hour. The temperature was lowered to room temperature and chloroform was added to the mixture. The mixture was separated with a separating funnel and the organic portion was collected and washed with water three times. The organic portion was concentrated and poured into a short plug of silica. The filtration cake was then washed with toluene and the filtrate was collected. The solvent in the filtrate was reduced to minimum and isopropanol was added. The solution was cooled to 0 °C for crystallization. The final precipitate was collected by suction filtrate and washed successively with isopropanol and methanol. The final product of **IDG-C20-Br** was a dark blue powder (678 mg , 10%) ^{1}H NMR (300 MHz, Chloroform-*d*) δ 9.01 (s, 2H), 7.01 (d, *J* = 8.3 Hz, 2H), 6.81 (d, *J* = 8.4 Hz, 2H), 3.94 (d, *J* = 5.5 Hz, 4H), 1.98 – 1.77 (m, 2H), 1.45 – 1.21 (m, 64H), 0.86 (t, *J* = 6.4 Hz, 12H). ^{13}C NMR (300 MHz, Chloroform-*d*) δ 186.56, 145.08, 144.00, 124.89, 121.58, 120.90, 117.53, 109.21, 72.05, 37.83, 37.83, 31.94, 31.25, 30.00, 29.69, 29.39, 26.81, 22.71, 14.13. Matrix

assisted laser desorption ionization time-of-flight mass spectrometry (MALDI-TOF-MS) was used to characterize the mass of the monomer **IDG-C20-Br**. The mass spectrum is shown in Figure S16. Calculated [$M+H^+$]: 1013.52; Found: 1013.37. (Figure S15 ,S16 & S117)

3.2. Synthesis of polymer **PIDG-T-C20** and **PIDG-BT-C20**

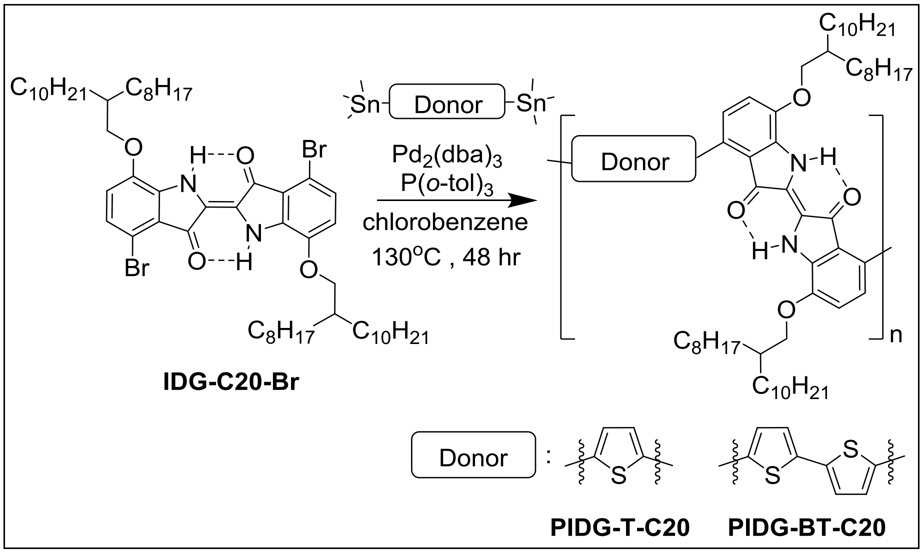

To a 25mL two-necked round-bottom flask, **4Br-7C20-IDG** (80 mg, 0.0790 mmol), P(*o*-tol)$_3$ (1.44 mg , 0.00473 mmol) and 5,5'-bis(trimethylstannyl)thiophene (32.4 mg, 0.0790 mmol) were added. One neck of the flask was fitted with a condenser and the other neck was stoppered with a rubber septum. The system was connected to argon atmosphere. The flask was then undergone a vacuum-purge cycle for three times and kept under argon atmosphere. Anhydrous chlorobenzene was added to dissolve all the solids before a solution of Pd$_2$(dba)$_3$ (1.08 mg , 0.00118 mmol) in chlorobenzene (1 mL) was added. The mixture was then stirred at 130 °C for 48 h. Upon cooling to room temperature, the viscous mixture was poured into methanol under stirring. The precipitate was collected and was purified by Soxhlet extraction using methanol, acetone and chloroform to afford polymer **PIDG-T-C20** as a dark blue film (68 mg, 92%). The synthesis of **PIDG-BT-C20** was carried out using a similar procedure using 5,5'-bis(trimethylstannyl)-2,2'-bithiophene as the donor building block and chlorobenzene as the final Soxhlet extraction solvent instead of chloroform. The final product obtained is a dark blue powder (72 mg, 90%).

4. Additional Data

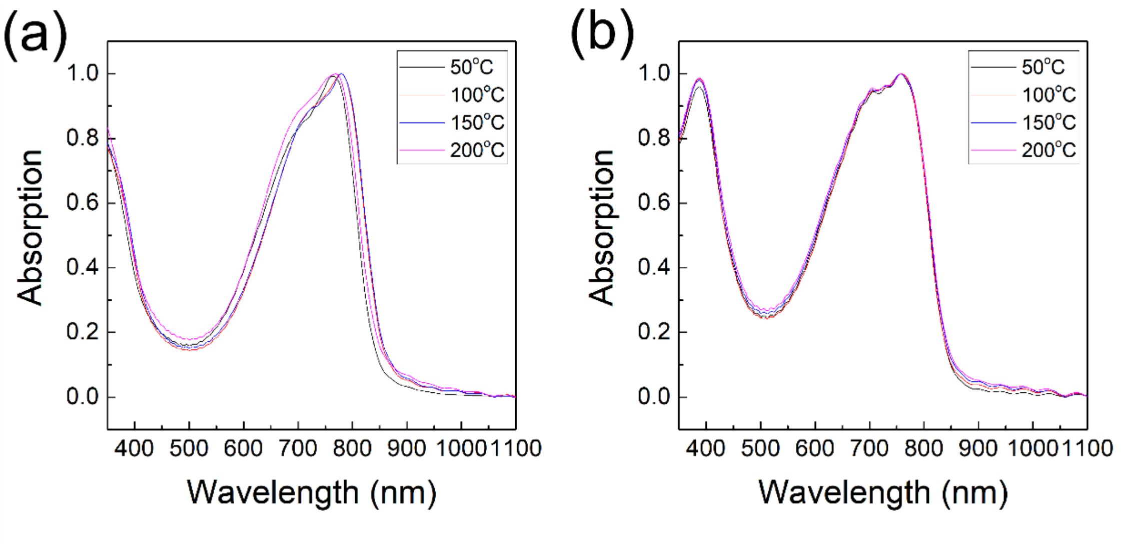

Figure S1 UV profiles of polymer thin films: (a) **PIDG-T-C20** and (b) **PIDG-BT-C20** annealed at 50, 100, 150, and 200 °C.

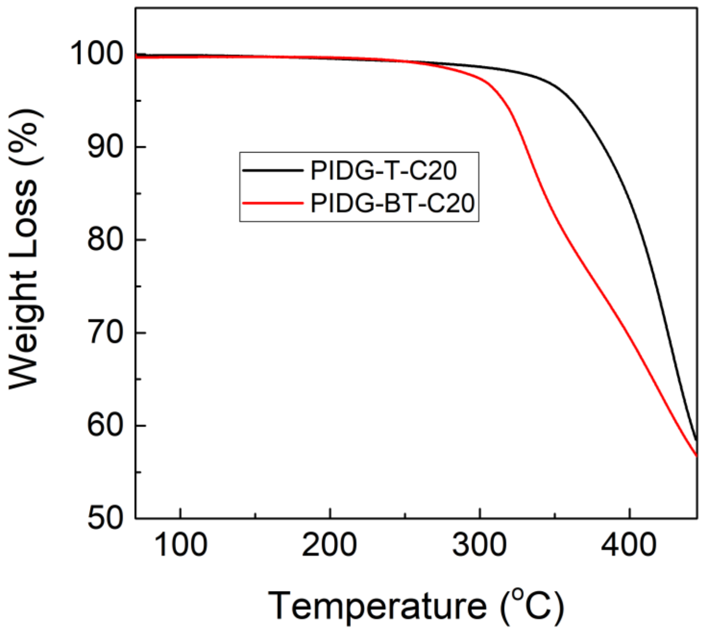

Figure S2 TGA graphs of **PIDG-T-C20** and **PIDG-BT-C20** at a heating rate of 20 °C min^{-1} under nitrogen.

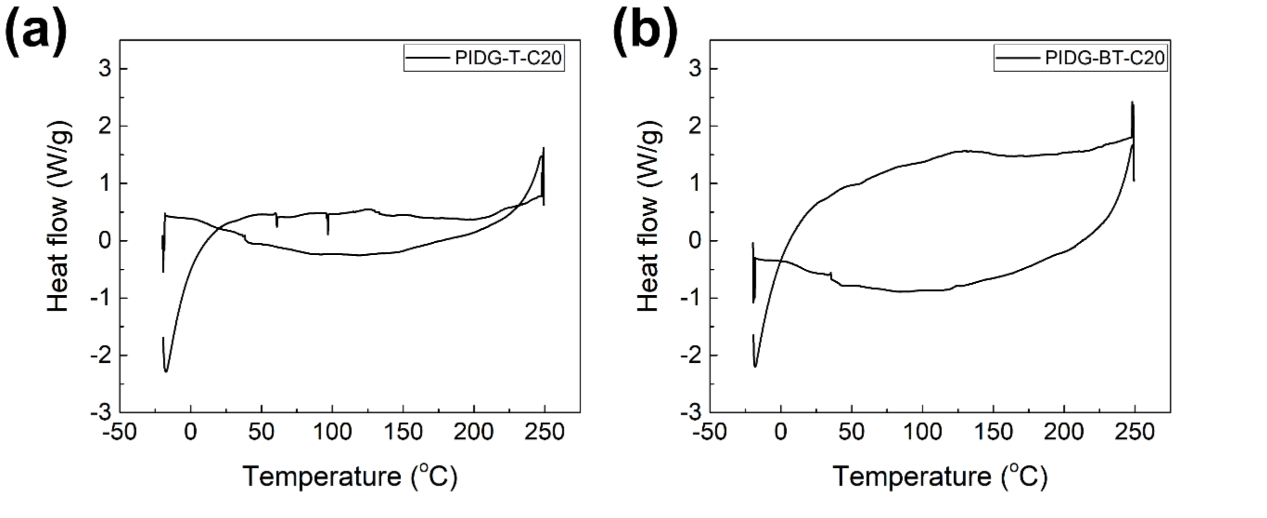

Figure S3 DSC thermograms of (a) **PIDG-T-C20** and (b) **PIDG-BT-C20** at a heating/cooling rate of 10 °C min^{-1} under nitrogen.

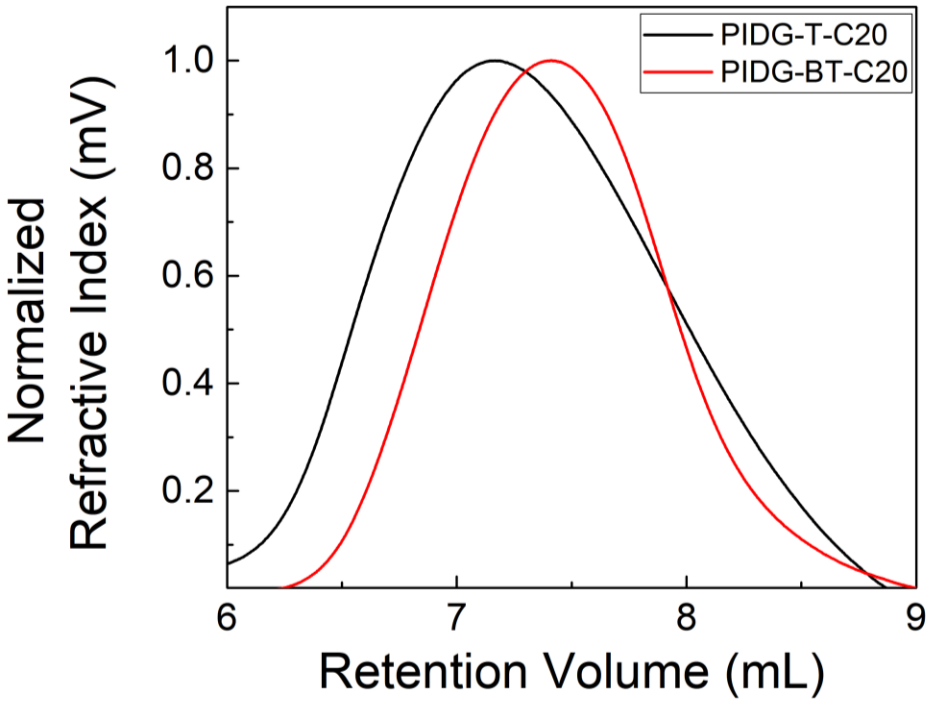

Figure S4 GPC chromatograms of indigoid polymers measured at 140 °C using 1,2,4-trichlorobenzene as eluent.

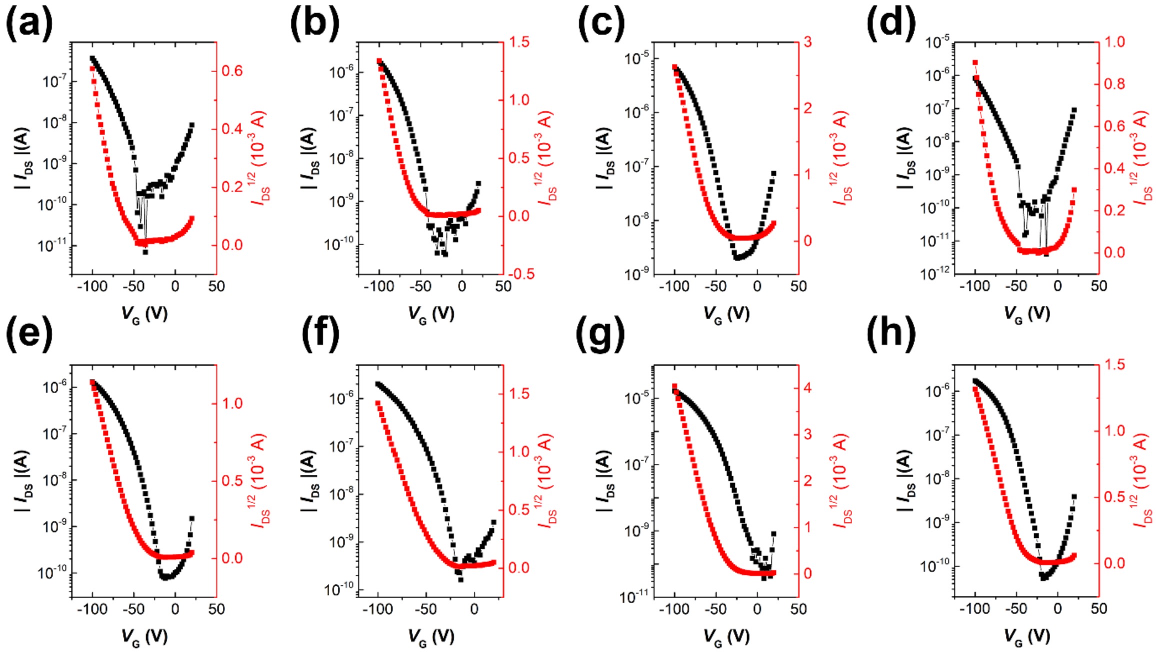

Figure S5 OFET transfer characteristics of **PIDG-T-C20** annealed at (a) 50 °C; (b) 100 °C; (c) 150 °C; (d) 200 °C and **PIDG-BT-C20** annealed at (e) 50 °C; (f) 100 °C; (g) 150 °C; (h) 200 °C with V_G step voltage = -10 V.

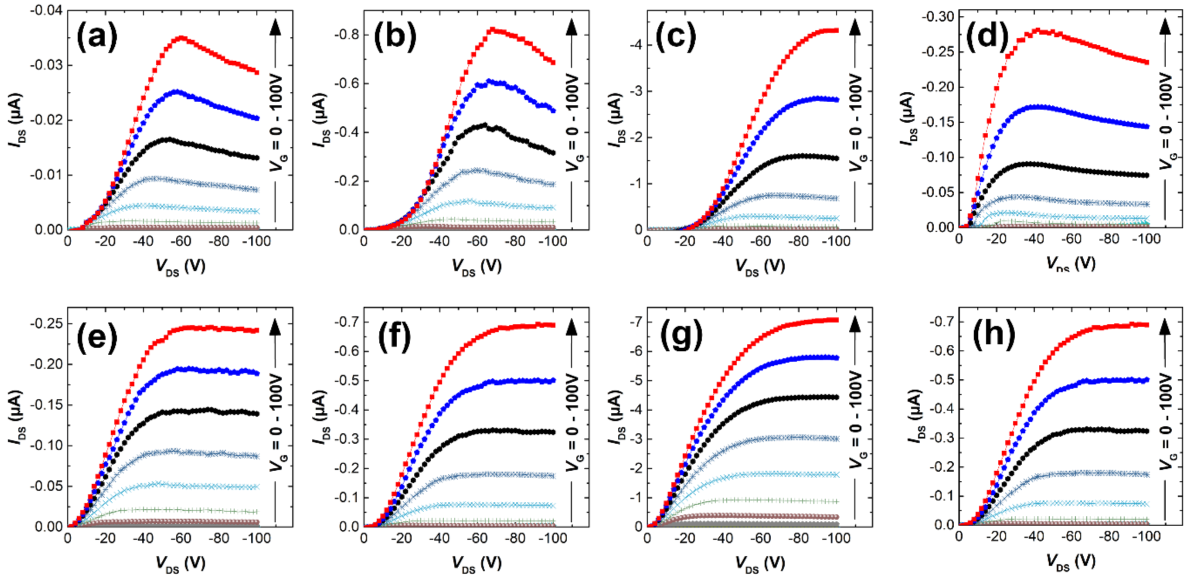

Figure S6 OFET output characteristics of **PIDG-T-C20** annealed at (a) 50 °C; (b) 100 ℃; (c) 150 °C; (d) 200 °C and **PIDG-BT-C20** annealed at (e) 50 °C; (f) 100 °C; (g) 150 °C; (h) 200 °C with V_G step voltage = -10 V.

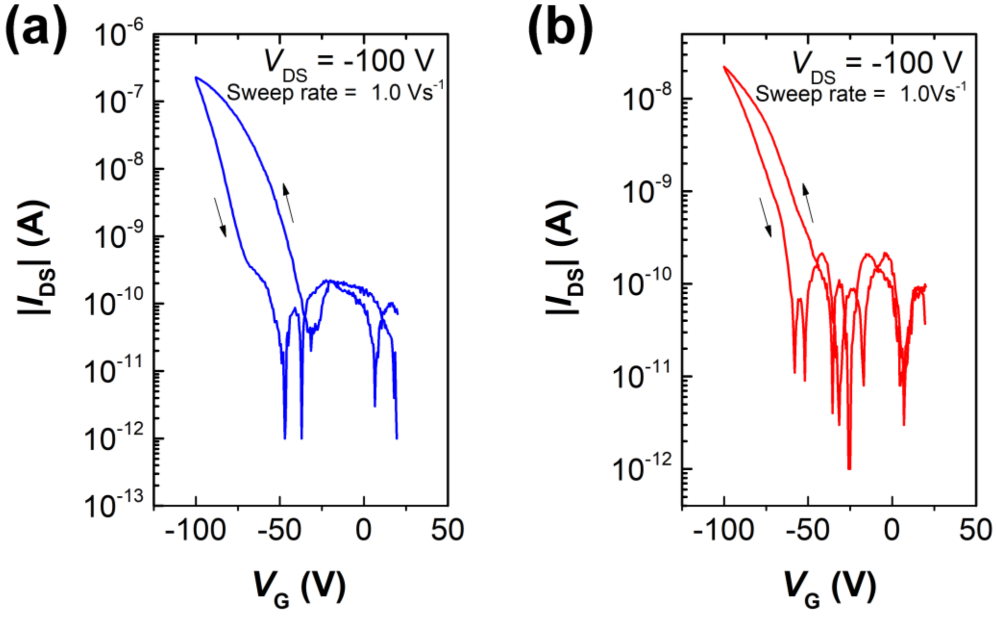

Figure S7 Hysteresis behaviors of the transfer characteristics of BGBC OFETs for (a) **PIDG-T-C20** and (b) **PIDG-BT-C20** films annealed at 150 °C by forward and backward V_G scan between +20 to -100 V at a sweep rate of 1.0 Vs^{-1}. The change in threshold voltage (ΔV_{th}) was found to be ΔV_{th} = 30.9V for **PIDG-T-C20** and ΔV_{th} = 7.90V for **PIDG-BT-C20**.

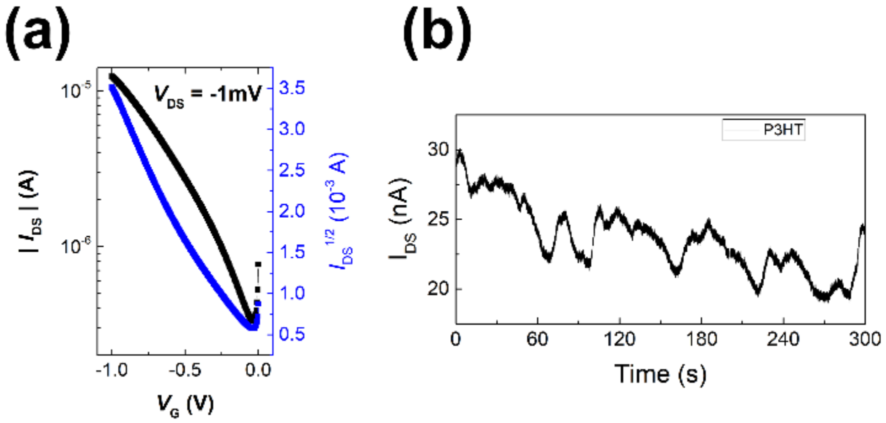

Figure S8 (a) Transfer characteristic of P3HT WGOFET without analyte; (b) the current vs time graph of P3HT based WGOFET showing a severe fluctuation.

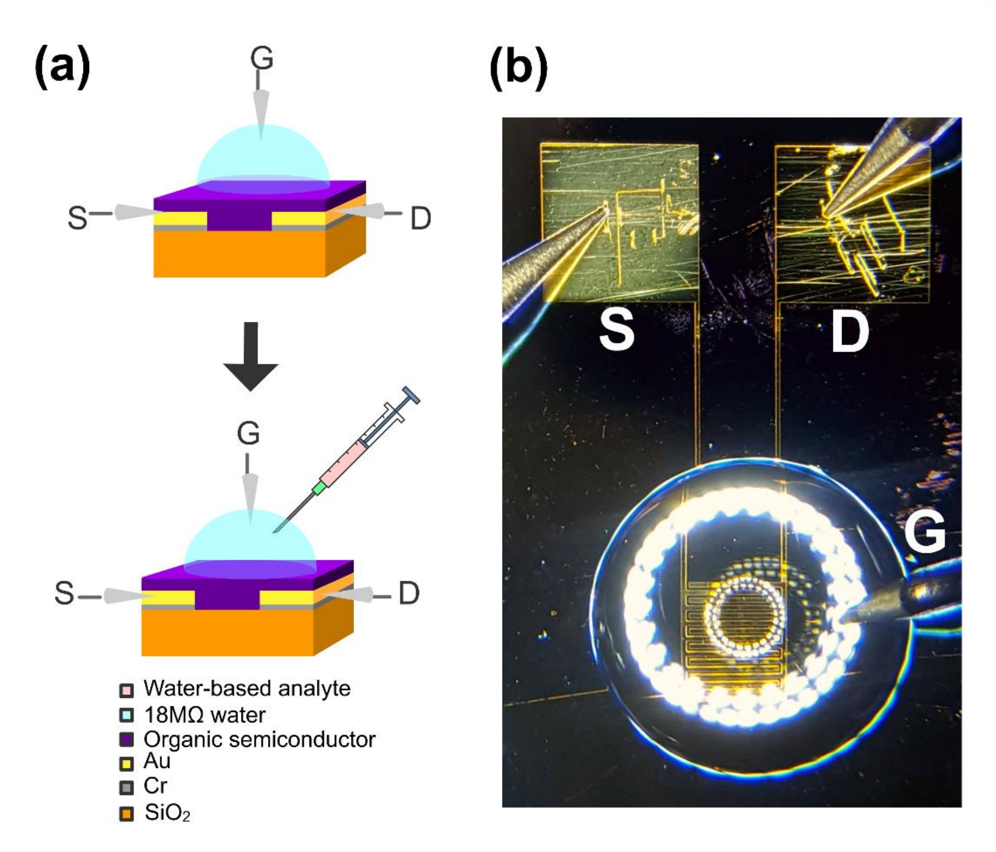

Figure S9 (a) Schematic diagram of analyte injection for a WGOFET sensor; (b) top view of a WGOFET sensor with interdigitated source (S) and drain (D) electrodes together with a gate (G) electrode contacting the water-dielectric droplet.

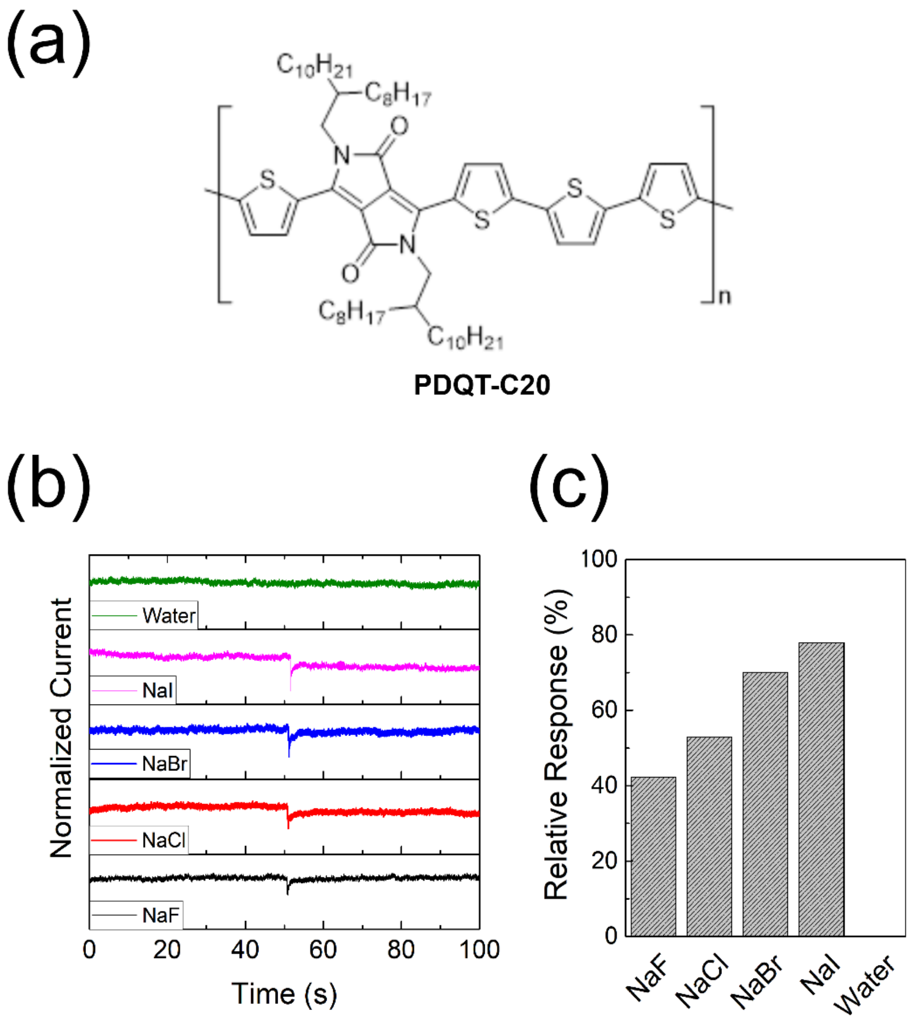

Figure S10 (a) Chemical structure of **PDQT-C20**; (b) current versus time graph (V_{DS} = -1 V and V_G = -1 mV) of sodium halide sensing experiment where 2.4 mM of 5μL analyte (NaF, NaCl, NaBr, NaI) was injected into the water-gate droplet on a **PDQT-C20** WGOFET device at around time = 50s; (c) histogram showing the response of PDQT-C20 towards different sodium halides at 2.4 mM (NaF = 42%, NaCl = 53%, NaBr = 70% and NaI = 78%).

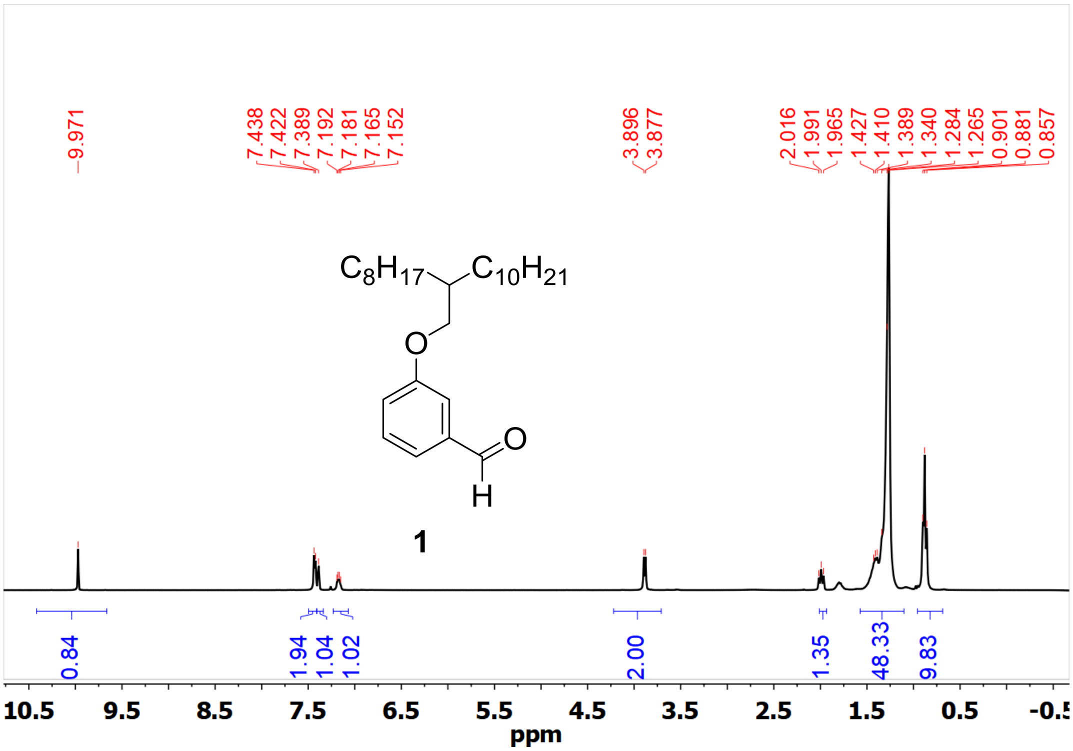

Figure S11 300 MHz ^{1}H NMR of Compound **1** in $CDCl_3$.

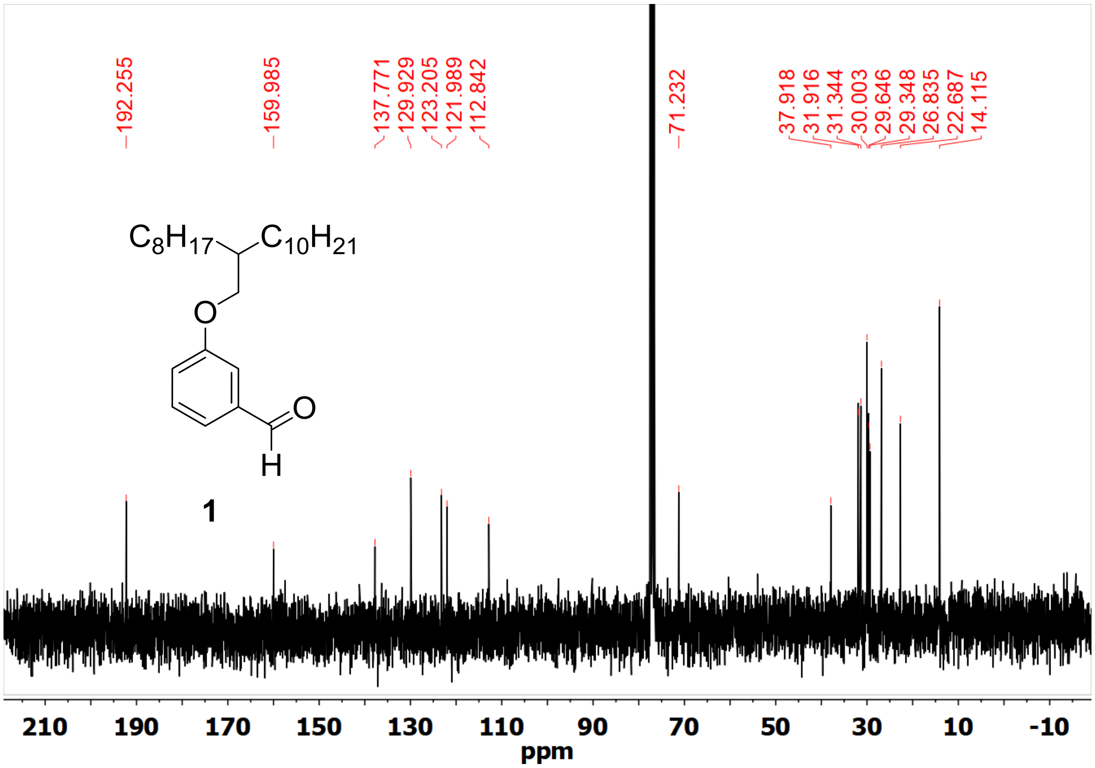

Figure S12 75 MHz ^{13}C NMR of Compound **1** in $CDCl_3$.

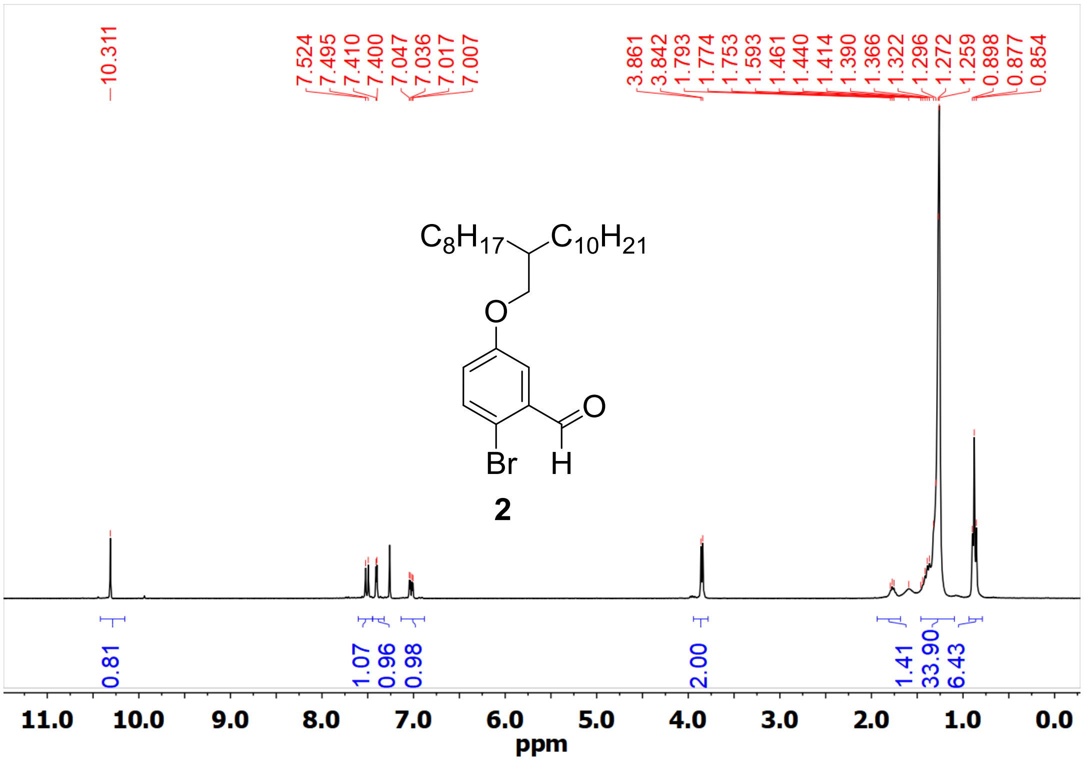

Figure S13 300 MHz ^{1}H NMR of Compound **2** in $CDCl_3$.

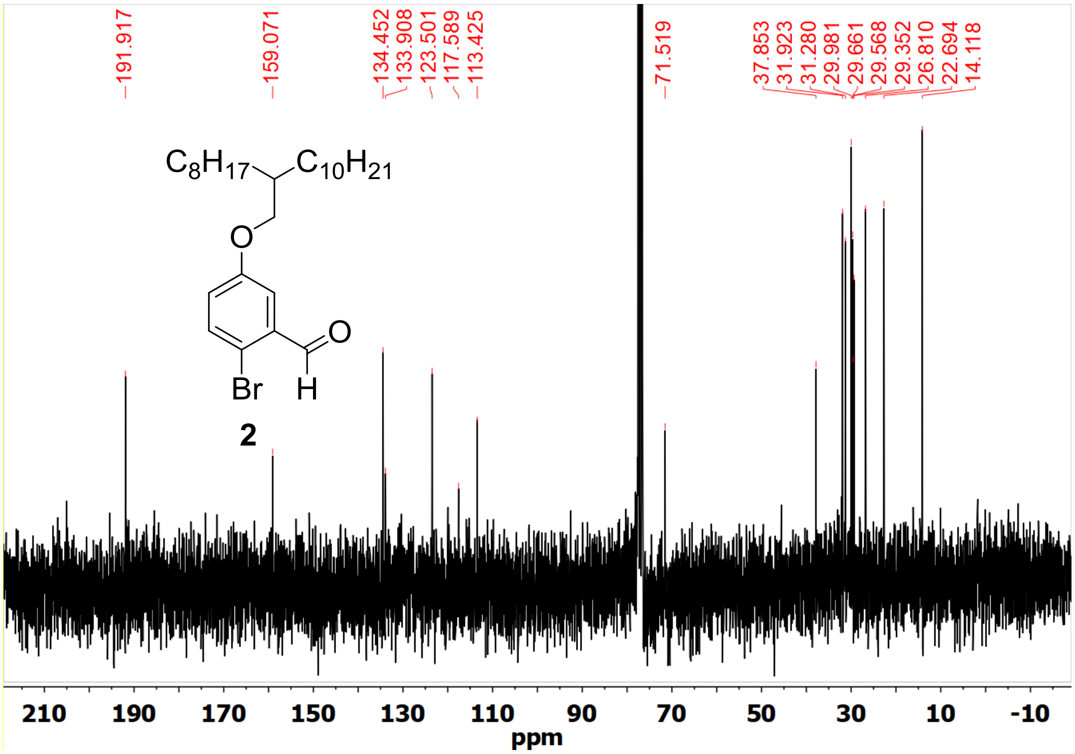

Figure S14 75 MHz ^{13}C NMR of Compound **2** in $CDCl_3$.

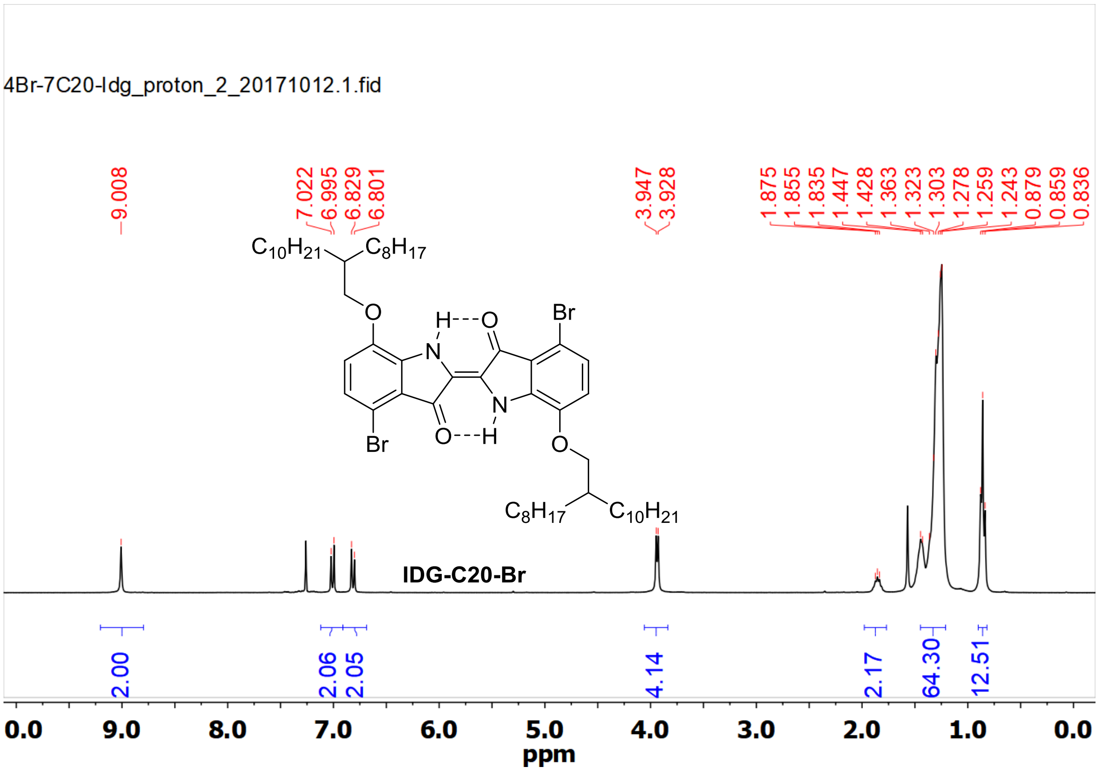

Figure S15 300 MHz ^{1}H NMR of **IDG-C20-Br** in $CDCl_3$.

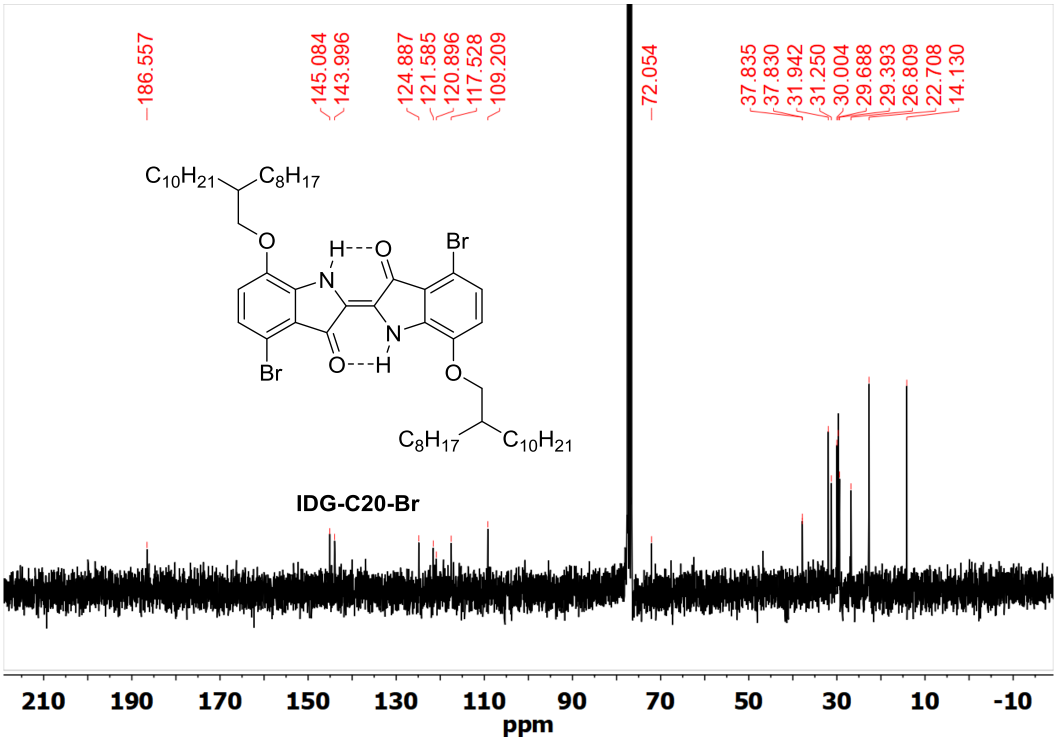

Figure S16 75 MHz ^{13}C NMR of **IDG-C20-Br** in $CDCl_3$.

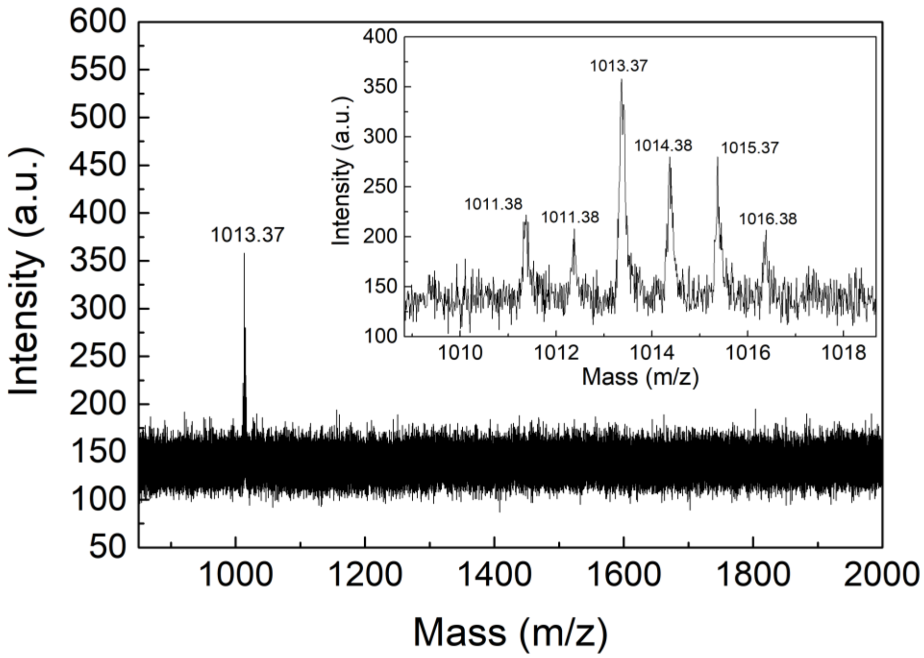

Figure S17 MALDI-ToF MS spectrum of **Br-IDG-C20**.

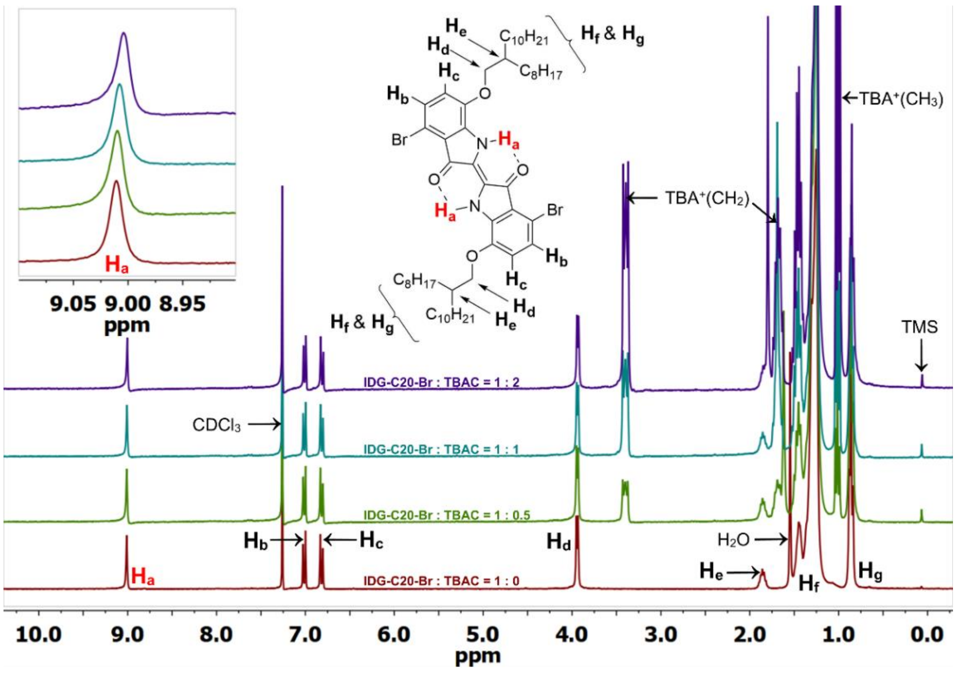

Figure S18 300 MHz ^{1}H NMR titration of **IDG-C20-Br** with TBAC (0 – 2 equiv) in $CDCl_3$.

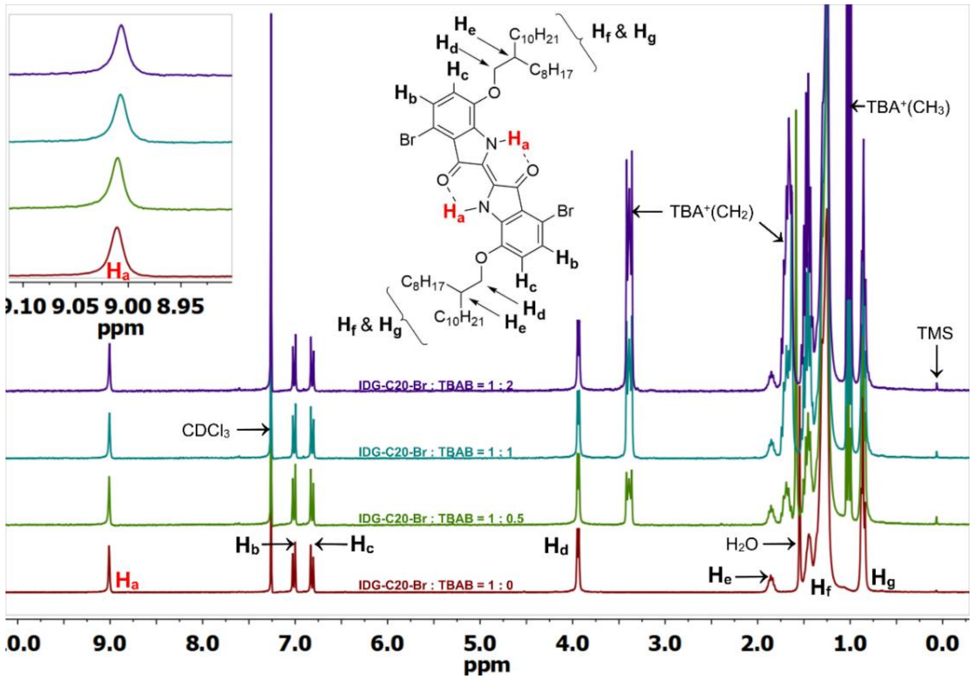

Figure S19 300 MHz ^{1}H NMR titration of **IDG-C20-Br** with TBAB (0 – 2 equiv) in $CDCl_3$.

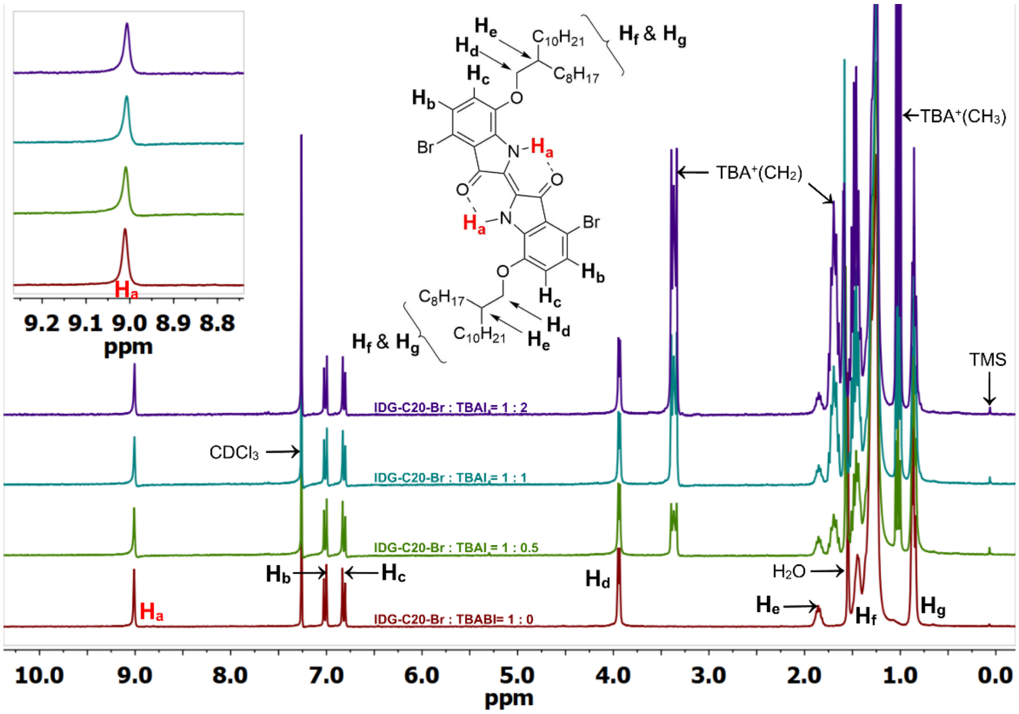

Figure S20 300 MHz ^{1}H NMR titration of **IDG-C20-Br** with TBAI (0 – 2 equiv) in $CDCl_3$.

Table S1 DFT calculation results of model compounds.

Models	Interatomic Distances (Å)			Charge	Spin
	[$H_a \cdots X$]	[$H_a \cdots N$]	[$H_a' \cdots N'$]		
**IDG**	---	1.02	---	0	doublet
**IDG-F**	1.00	1.66	1.02	-1	singlet
**IDG-Cl**	2.07	1.06	1.02	-1	singlet
**IDG-Br**	2.14	1.07	1.02	-1	singlet
**IDG-I**	2.67	1.04	1.02	-1	singlet

5. Reference

1 S. Chen, B. Sun, W. Hong, H. Aziz, Y. Meng and Y. Li, *J. Mater. Chem. C*, 2014, **2**, 2183–2190.

2 J. Pommerehne, H. Vestweber, W. Guss, R. F. Mahrt, H. Bässler, M. Porsch and J. Daub, *Adv. Mater.*, 1995, **7**, 551–554.
